# Rice *gs3* allele and low-nitrogen conditions enrich rhizosphere microbiota that mitigate methane emissions and promote beneficial crop traits

**DOI:** 10.1093/ismejo/wraf284

**Published:** 2025-12-29

**Authors:** Youngho Kwon, Jisu Choi, Sung Hoon Kim, Pil Joo Kim, So-Myeong Lee, Jin-Kyung Cha, Hyeonjin Park, Ju-Won Kang, Sumin Jo, Youn-Sig Kwak, Dajeong Kim, Woo-Jae Kim, Jong-Hee Lee, Choong-Min Ryu

**Affiliations:** Field crop research division, National Institute of Crop and Food Science, RDA, Miryang 50424, Gyeongsangnamdo, South Korea; Field crop research division, National Institute of Crop and Food Science, RDA, Miryang 50424, Gyeongsangnamdo, South Korea; Field crop research division, National Institute of Crop and Food Science, RDA, Miryang 50424, Gyeongsangnamdo, South Korea; Division of Applied Life Science (BK21+ Program), Gyeongsang National University, Jinju 52828, Gyeongsangnamdo, South Korea; Field crop research division, National Institute of Crop and Food Science, RDA, Miryang 50424, Gyeongsangnamdo, South Korea; Field crop research division, National Institute of Crop and Food Science, RDA, Miryang 50424, Gyeongsangnamdo, South Korea; Field crop research division, National Institute of Crop and Food Science, RDA, Miryang 50424, Gyeongsangnamdo, South Korea; Field crop research division, National Institute of Crop and Food Science, RDA, Miryang 50424, Gyeongsangnamdo, South Korea; Field crop research division, National Institute of Crop and Food Science, RDA, Miryang 50424, Gyeongsangnamdo, South Korea; Department of Plant Medicine, Gyeongsang National University, Jinju 52828, Gyeongsangnamdo, South Korea; Molecular Phytobacteriology Laboratory, Infectious Disease Research Center, KRIBB, Daejeon 34141, South Korea; Field crop research division, National Institute of Crop and Food Science, RDA, Miryang 50424, Gyeongsangnamdo, South Korea; Field crop research division, National Institute of Crop and Food Science, RDA, Miryang 50424, Gyeongsangnamdo, South Korea; Molecular Phytobacteriology Laboratory, Infectious Disease Research Center, KRIBB, Daejeon 34141, South Korea

**Keywords:** methane emissions, microbiome, nitrogen use efficiency, rice breeding, sustainable agriculture

## Abstract

Methane emissions from rice paddies represent a critical environmental concern in agriculture. Although genetic strategies for mitigating emissions have gained attention, the specific microbial and molecular mechanisms remain underexplored. Here, we investigated how the *gs3* loss-of-function allele in the near-isogenic rice line Milyang360 modulates rhizosphere and endosphere microbial communities under distinct nitrogen regimes. Field experiments revealed that Milyang360 consistently reduced methane emissions compared with its parental line Saeilmi particularly under low-nitrogen conditions. Integrated plant transcriptomic and rhizosphere metagenomic analyses, including the reconstruction of Metagenome-Assembled Genomes, demonstrated that the *gs3* allele upregulated genes related to root hair elongation and promoting microbial nitrogen fixation. This physiological change limited substrate availability for methanogens and facilitated the colonization by beneficial microorganisms. Consequently, we observed a functional shift in the microbiome, characterized by the enrichment of methanotrophs and nitrogen-fixing bacteria. This microbial restructuring was most prominent under low-nitrogen conditions, indicating a strong genotype by environment interaction. Our findings highlight the *gs3* allele’s dual role in reducing methane emissions and improving nitrogen use efficiency by recruiting a beneficial microbiome. Our study provides a clear mechanistic link between a plant gene and rhizosphere ecology, offering a promising genetic target for developing sustainable, low emission rice cultivars.

## Introduction

Methane (CH₄) emissions from rice paddies represent a significant source of anthropogenic greenhouse gases, accounting for ~7%–17% of total atmospheric methane [1, 2]. These emissions are driven primarily by the anaerobic decomposition of organic matter in flooded soils, a process governed by complex interactions among plant physiology, soil microbial communities, and nutrient management. As rice is the staple food for over half of the world’s population, developing cultivation practices that mitigate environmental impact while sustaining high yields is a pressing global challenge [3]. Within the unique ecosystem of flooded rice paddies, the rhizosphere plays a pivotal role in regulating methane fluxes [4]. Methanogens, predominantly anaerobic archaea thriving in oxygen-depleted soil, utilize plant-derived organic substrates to produce methane [1, 5]. Conversely, methane-oxidizing bacteria, or methanotrophs, mitigate emissions by converting methane to carbon dioxide [6, 7]. The balance between these microbial guilds is heavily influenced by plant genetic factors and environmental conditions, such as nutrient availability. Therefore, a mechanistic understanding of how plant genetics and agronomic practices shape these interactions is vital for mitigating methane emissions and achieving sustainable rice production.

Recent advances in rice genetics have opened new avenues for methane mitigation [8–10]. The development of near-isogenic lines such as Milyang360, provides a powerful tool to elucidate how specific rice genes modulate methane emissions. Milyang360, derived from the Saeilmi variety, is distinguished by a natural loss-of-function allele of *gs3*, a gene traditionally associated with grain size variation [11, 12]. Previous work has suggested that the *gs3* allele can mitigate methane emissions by altering photosynthate allocation, partitioning more carbon to the grains and less to the roots [9]. However, previous study focused on the macro-level outcome and did not resolve the precise molecular and microbial mechanisms at the plant- microorganism interface. It remained unknown how *gs3* driven changes in root gene expression actually restructure the rhizosphere microbiome to regulate the balance between methanogenesis and methanotrophy.

Nitrogen availability is another critical factor influencing methane emissions from paddy fields. Nitrogen fertilization can enhance plant growth and root biomass, increasing organic carbon inputs to the soil and stimulating methanogenic activity [13–15]. Among the components of nitrogen fertilizer, ammonium can also inhibit methane consumption by methanotrophs, which compete for methane monooxygenase [16]. While the importance of nitrogen management is well recognized, the genetic factors that dictate plant and microbial responses to varying nitrogen regimes are underexplored.

**Figure 1:**
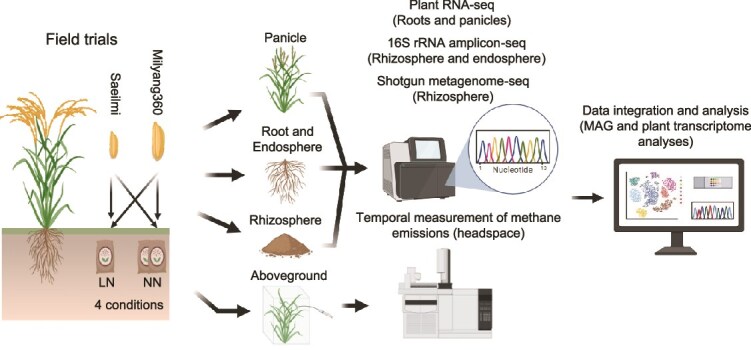
Experimental workflow and analytical scheme. Two rice varieties Milyang360 and Saeilmi were cultivated under low nitrogen (LN, 45 kg·N /ha) and normal nitrogen (NN, 90 kg·N/ha) treatments. Methane emissions were periodically measured throughout the cultivation period. Plant samples (panicles and roots) were collected for transcriptome analysis via RNA-seq, while both rhizospheric and endospheric samples were analyzed for microbial community structure (16S rRNA gene amplicon sequencing) and only rhizospheric samples were analyzed for functional genes (shotgun metagenomic sequencing). Metagenomic data were processed for MAG reconstruction and functional annotation. All datasets were integrated for comprehensive analysis of plant- microorganism-methane interactions.

This study aims to fill this critical mechanistic gap left by our previous report [9]. We move beyond the known phenomenon to test two underlying mechanisms. We hypothesized that *gs3* driven changes in carbon transport and symbiotic signaling pathways would lead to a restructured microbial community. Specifically, we test two new hypotheses (i) whether the *gs3* allele actively enriches beneficial methanotrophs and nitrogen-fixers rather than only suppressing methanogens; and (ii) this is driven by the plant’s upregulation of symbiotic signaling and root development genes, not just changes in carbon transport. To investigate these mechanisms, we employed a multi-omics approach, integrating field-level measurements with plant transcriptomics and rhizosphere metagenomics. We reconstructed metagenome-assembled genomes (MAGs) to link specific microbial lineages directly to their functional roles in methane and nitrogen cycling under low-nitrogen (LN) and normal nitrogen (NN) conditions (Fig. 1). The *gs3* allele acts synergistically with low nitrogen availability to significantly reduce methane emissions. This effect is achieved through a dual mechanism, the downregulation of root carbon transporters which limits substrate supply to methanogens and the enrichment of beneficial microorganisms including methanotrophs and nitrogen-fixing bacteria. Our genome-resolved analysis provides a clear mechanistic link between specific plant genes such as the root hair elongation, the functional reorganization of the rhizosphere microbiome, and the mitigation of greenhouse gas emissions, offering a robust genetic foundation for developing low emission, high yield rice varieties for a sustainable future.

## Materials and methods

### Experimental design and field trials

The field experiments were conducted as previously described [9], Miryang, South Korea (35° 29′ 32.2872″ N, 128° 44′ 32.1972″ E) and Jinju, South Korea (35° 9′ 12.14268″ N, 128° 6′ 3.73284″ E), from 2022 to 2023. Before transplanting, standard tillage was performed, and no straw or organic material was added to the fields. Two rice varieties, Milyang360 and Saeilmi, were used in this study. Seedlings were grown for 30 d before transplanting to both sites on June 5 at a planting density of 30 × 15 cm. The experimental plots, each measuring 8 × 70 m, were arranged in triplicate. A chemical fertilizer with a nutrient composition of N-P_2_O_5_-K_2_O (21–17-17) was applied at a total nitrogen rate of 90 kg ha − 1 throughout the growing season. Nitrogen was distributed as follows: 54 kg ha − 1 was applied as a basal treatment before transplanting, 18 kg ha—1 was administered 20 days after transplanting (DAT), and the final 18 kg ha—1 was applied at 65 DAT under normal nitrogen conditions. In the plots designated for low nitrogen treatment, only half of the nitrogen used in the normal plots was applied. These normal and low N plots have been maintained under consistent management over the past 20 years. Field management included continuous flooding until 110 DAT, with water levels maintained 5–7 cm above the soil surface throughout the rice growing period to ensure proper water availability.

### Measurement of methane emissions

A closed chamber experiment was conducted to measure methane emissions from rice crops cultivated in Miryang and Jinju as previously described [9]. Similarly, a separate closed chamber experiment was conducted in Jinju over two years to assess emissions under both low and normal nitrogen conditions. Gas samples were collected from each treatment at 15-min intervals over a 30-min duration using 50 ml plastic syringes equipped with three-way stopcocks. The sampling was performed in three glass chambers (62 cm in diameter and 112 cm in height) installed in each plot. The chambers, positioned on flooded soil to enclose eight rice hills each, had four holes at the bottom to maintain a consistent water level of 5–7 cm above the soil water interface.

Sampling was conducted at 10:00 a.m. local time, once a week throughout the rice-growing season, to calculate the daily methane flux. The chambers were fitted with fans to circulate air and thermometers to monitor air temperature during sampling. The chambers remained open except when the gas samples were collected, and the samples were immediately transferred to air-evacuated glass vials for laboratory analysis. Methane concentrations were measured using an Agilent 7890A gas chromatograph equipped with a stainless-steel Porapak NQ column (Q80–100 mesh, cat. no. 1002–11 105) and a flame ionization detector. The column, injector, and detector were maintained at 80, 100, and 110°C, respectively. Helium was the carrier gas, and hydrogen was the burning gas. Methane flux was calculated using the following equation:


(1) \begin{equation*} \mathrm{F}=\left(\mathrm{V}/\mathrm{A}\right)\times \left(\Delta /\Delta \mathrm{t}\right)\times \left(273/\mathrm{T} \right) \end{equation*}

The equation considers factors such as methane flux (*F*, mg/m^2^/h), chamber volume (*V*), chamber area (*A*), rate of methane accumulation, and absolute temperature during gas sampling (*T*). If the data points deviated significantly from the regression line or if the correlation coefficient was not significant at the 95% confidence level, the sample was either recalculated without outliers or discarded.

The total methane flux for the cropping period was calculated using the following equation:


(2) \begin{equation*} \mathrm{Total}\ \mathrm{methane}\ \mathrm{flux}={\sum_i^n}\left( Ri\times Di\right) \end{equation*}

where *Ri​* is the methane emission rate in g/m^2^/d for each sampling interval *i, Di*​ represents the number of days within each interval, and *n* is the total number of sampling intervals. This calculation provides the overall methane emission rate.

### 16S rRNA gene amplicon, metagenome sequencing and data analysis of the rhizosphere and endosphere microbiota for Milyang360 and Saeilmi

Samples represent 24 treatments and were collected at the key growth stages of tillering, heading, and grain filling in Miryang experimental field. DNA was extracted from both soil and endophytic samples using a FastDNA Spin Kit (MP Bio) according to the manufacturer’s protocol. To separate the rhizosphere samples, roots were shaken vigorously to remove loosely attached bulk soil. The soil that remained tightly adhered to the root surface, ~1–2 mm thick, was collected using sterile forceps and considered the rhizosphere sample. For the endosphere samples, the remaining roots were processed immediately. First, roots were washed with sterile phosphate-buffered saline (PBS) three times to remove any remaining soil particles. To eliminate epiphytic microorganisms, the washed roots were surface-sterilized by sequential immersion in 70% ethanol for 1 min, followed by 1% sodium hypochlorite (bleach) for 3 min, and then 70% ethanol for 30 s. Finally, the surface-sterilized roots were rinsed five times with sterile deionized water to remove any residual sterilization agents. These roots were then used for endophytic DNA extraction [17]. The extracted DNA was then amplified using polymerase chain reaction (PCR) with universal 16S ribosomal RNA gene primers (Bioneer), specifically targeting the bacterial 16S rRNA gene sequence in the V3–V4 region. The amplified DNA was then purified, quantified, pooled, and prepared for emulsion PCR using Herculase II Fusion DNA Polymerase and Nextera XT Index Kit V2 on a MiSeq System (Illumina). Following PCR, the amplicons were purified. A second PCR (index PCR) was performed to attach Illumina sequencing adapters and dual-indices using the Nextera XT Index Kit. The indexed amplicons were purified again, and their final quality and concentration were assessed using Agilent 2100 Bioanalyzer and Qubit fluorometer. The qualified libraries were pooled in equimolar concentrations and sequenced on a MiSeq system (Illumina).

**Figure 2:**
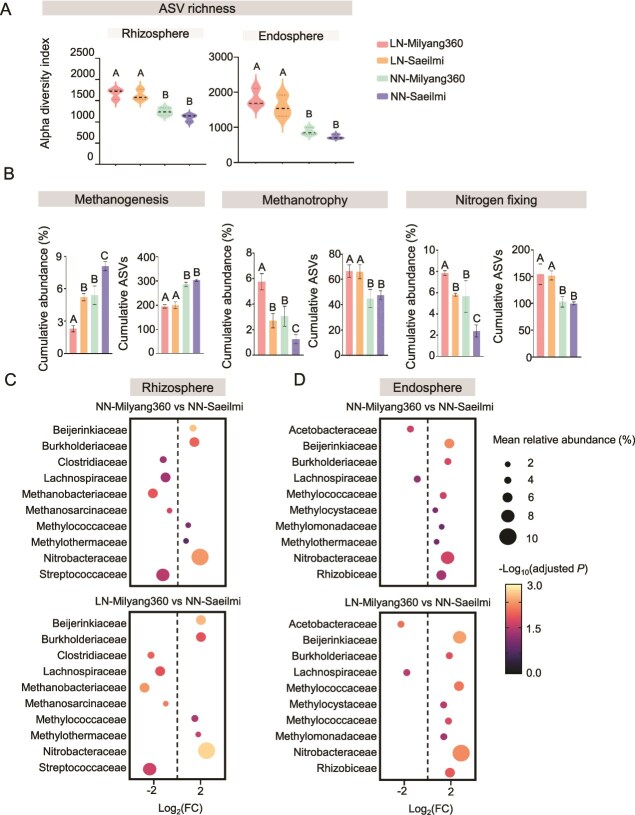
Alpha diversity, cumulative abundance, ASVs, and comparison of the enrichment microorganisms between LN-Milyang360 and NN-Saeilmi. (A) Microbial alpha diversity metrics (ASV richness) for rhizosphere and endosphere samples. Letters indicate statistically significant differences between treatments (*P* < .05; Tukey’s HSD test). (B) Cumulative abundances and ASV counts for key functional guilds inferred using FAPROTAX, including methanogenesis, methanotrophy, and nitrogen fixation. Letters indicate statistically significant differences between treatments (*P* < .05; Tukey’s HSD test). Error bars represent standard error. (C and D) Differential abundance of microbial families in the rhizosphere and endosphere. X-axis represents the Log₂ fold change (Log₂FC). Positive values indicate enrichment in LN-Milyang360 or NN-Milyang360, while negative values indicate enrichment in NN-Saeilmi. Bubble size corresponds to the mean relative abundance (%) of that family across the samples. Bubble color represents the statistical significance, showing the –Log_10_(adjusted *P*-value).

The raw sequencing data were processed using the QIIME2 (v2022.2) pipeline. Paired-end reads were first joined, and then quality-filtered and denoised into amplicon sequence variants (ASVs) using the DADA2 plugin (qiime dada2 denoise-paired). This process corrects for sequencing errors and generates a feature table of exact sequence variants. Chimeric sequences were identified and removed during this step. Taxonomic classification was assigned to each ASV using a pre-trained Naive Bayes classifier based on the SILVA (v138) 16S rRNA gene database, clustered at 99% similarity. ASVs identified as mitochondria or chloroplasts were removed from the feature table. Alpha diversity metrics (ASV Richness) were calculated to assess within sample diversity. Beta diversity was calculated using Bray–Curtis dissimilarity and visualized via principal coordinates analysis (PCoA). Permutational multivariate analysis of variance (PERMANOVA) was used to test for significant differences in community structure among treatment groups. To infer putative ecological functions from the 16S rRNA gene taxonomic data, we used FAPROTAX (v.1.2.4) [18]. This tool maps prokaryotic taxa (ASVs) to established metabolic functions. The functional groups relevant to this study (e.g. methanogenesis, methanotrophy, nitrogen fixation) were aggregated to calculate the cumulative abundance (relative abundance %) and ASV counts (richness) for each functional guild per sample (Fig. 2B). Differentially abundant microbial families (adjusted *P* < .05) were visualized using bubble plots (Fig. 2C and D). In these plots, the x-axis represents the log₂ fold change between conditions, while the size and color of each bubble correspond to the mean relative abundance (%) and -log₁₀ (adjusted *P-*value) of that family across all samples.

For metagenome analysis, rhizosphere samples were collected in Miryang experimental field after heading stage, paired-end (2 × 150 bp) sequencing was performed on a NovaSeq 6000 System (Illumina). Raw sequencing reads were subjected to stringent quality control using Trimmomatic v0.39 [19]. Adapter sequences were removed, and low-quality bases were trimmed using a sliding window approach (4-base window, average Phred score < 20). Reads shorter than 50 bp were discarded. To remove host-derived sequences, the quality-controlled reads were mapped against the *Oryza sativa japonica* reference genome (IRGSP-1.0) using Bowtie2 v2.4.1 with default parameters. Reads that did not map to the host genome were retained as microbial reads for downstream analysis. The quality of the final clean reads was verified using FastQC v0.11.9 [19]. The high-quality, nonhost reads from all samples were co-assembled into a single comprehensive set of contigs using MEGAHIT v1.2.9 with the -meta-large preset option, which is optimized for complex metagenomes [20, 21]. The quality of the final assembly was evaluated using QUAST v5.0.2 to calculate metrics such as N50, number of contigs, and total assembly length.

Protein-coding genes (ORFs) were predicted from contigs longer than 500 bp using Prodigal v2.6.3 in meta mode (−p meta) [22]. The resulting protein sequences were annotated against the KEGG Orthology (KO) database using KofamKOALA (v2022-03-01) with HMMER3 and adaptive score thresholds for each KO group. To calculate the relative abundance of functional genes, we used a TPM (Transcripts Per Million)-based normalization scheme, which accounts for both gene length and sequencing depth [23]. Specifically, clean reads from each sample were mapped back to the assembled contigs using Bowtie2 v2.4.1, and read counts per gene were converted to TPM as


$$ {\mathrm{TPM}}_i=\frac{\frac{R_i}{L_i}}{\sum_{j=1}^n\frac{R_j}{L_j}}\times{10}^6 $$

Where ${R}_i$is the number of reads mapped to gene $i$ and ${L}_i$is the length (in kilobases) of gene $i$. This method allows for accurate cross-sample comparisons of gene abundance while correcting for gene length bias. To further validate KO assignments, we cross-checked the annotations using eggNOG-mapper v2.1.7 [24] and Pfam-A (v35.0) HMM profiles via HMMER3 [25], ensuring functional consistency and minimizing annotation artifacts. For community-level analysis, gene-level TPM values were summed by KO categories, and visualized as Z-score normalized heatmaps across treatment groups. Z-score transformation was performed per gene across all samples to highlight relative enrichment trends.


$$ {Z}_{ij}=\frac{TPM_{ij}-{\mu}_i}{\sigma_i} $$

In this equation, ${\mu}_i$ denotes the average TPM value of gene, $i$, and ${\sigma}_i$denotes the standard deviation of TPM values for that gene, both calculated across all samples. High-quality MAGs (≥60% completeness, ≤10% contamination) were reconstructed using MetaBAT2 followed by CheckM for quality control. The taxonomic classification of each high-quality MAG was determined using the Genome Taxonomy Database Toolkit (GTDB-Tk v2.4.0).

**Figure 3:**
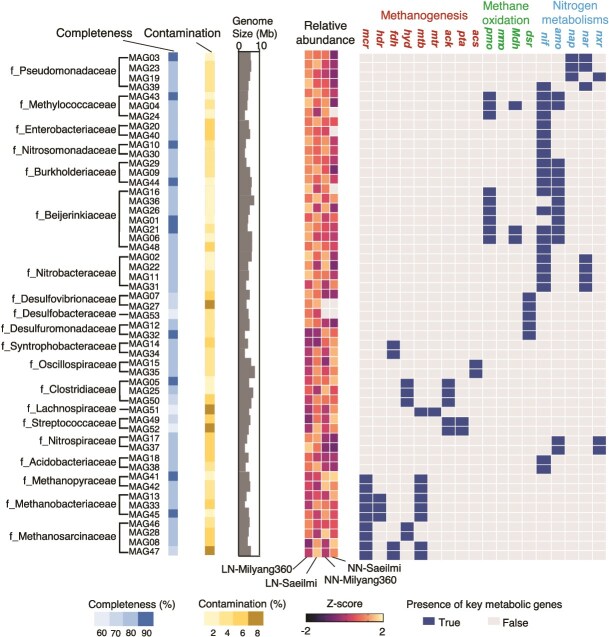
Functional and taxonomic characterization of metagenome-assembled genomes (MAGs) from the rice rhizosphere. The 52 MAGs are listed by their taxonomic family determined by GTDB-Tk. Adjacent bar plots showing completeness, contamination, and estimated genome size. Relative abundance across the four treatment conditions visualized as a Z-score normalized heatmap. The presence (filled box) or absence (empty box) of metabolic key genes.

The relative abundance of each MAG in each sample was calculated by mapping the clean reads from each sample to the MAG sequences using Bowtie2 v2.4.1. The abundance was expressed as the mean coverage of the MAG, normalized by the total number of microbial reads in the sample. To visualize the functional potential of each MAG (Fig. 3), we analyzed the protein-coding genes predicted by Prodigal. Based on the KO annotations, we screened for the presence or absence of key genes involved in the metabolic pathways such as methanogenesis, methanotrophy, and nitrogen metabolisms.

### Quantification of methanogens and methanotrophs in the rhizosphere and endosphere

Soil microbial DNA was extracted from the soil (0.5 g) and root samples for both rhizosphere and endophytic analyses. Soil DNA was extracted using a FastDNA Spin Kit for Soil (MP Bio). The gene copy numbers of methanogens (*mcrA*) and methanotrophs (*pmoA*), were quantified using real-time PCR and a QuantStudio 5 system (Thermo Fisher Scientific).

Absolute quantification of both the soil and root samples was performed using qPCR. To generate a standard curve, a 1/10 serial dilution of the initial standard mass particle solution was used, and the template was amplified to establish a threshold cycle (Ct) for known concentrations. The obtained correlation coefficients (*R* [2]) were greater than 0.99, indicating reliable quantification. Details of the primers used for the analysis are described in Supplementary Tables 4 and 5.

### Gene expression analysis

Total RNA was isolated from different plant tissues using an RNeasy Kit (Qiagen) according to the manufacturer’s protocol. Complementary DNA (cDNA) was synthesized using the ProtoScript II First Strand cDNA Synthesis Kit (NEB). Real-time PCR was performed using the amfiSure qGreen qPCR Master Mix (2X) with low ROX (GenDEPOT) on a QuantStudio 5 system (Thermo Fisher Scientific) for 40 cycles. Gene expression was quantified using real-time PCR, and transcript levels were normalized to the expression of the rice *Actin1* gene. The specific primer sequences used for real-time PCR are described in detail in Supplementary Table 4.

### Assessment of soil physicochemical characteristics and measurement of rice agronomic traits

Experimental plots located at the Department of Southern Area Crop Science, National Institute of Crop Science, Rural Development Administration (35° 29′ 32.2872″ N, 128° 44′ 32.1972″ E) have been maintained for 20 years under two nitrogen levels: normal (9 kg per 10 acres) and low (4.5 kg per 10 acres). These plots were used to assess soil physicochemical properties, including soil pH, electrical conductivity, total nitrogen (N), extractable phosphorus (P), and potassium (K) (Supplementary Table 2). Soil pH was determined using a 1:2.5 soil-to-deionized water suspension, and electrical conductivity was measured using a 1:5 soil-to-deionized water suspension. The Kjeldahl method was used for total nitrogen analysis, whereas the Walkley and Black wet oxidation methods were used to measure total oxidizable organic carbon. The resulting organic carbon content was multiplied by 1.72 to estimate the organic matter content. The extractable phosphorus and potassium contents were assessed using the Egner–Riehm extraction method with a solution of 0.1 M ammonium lactate and 0.4 M acetic acid (pH 3.65–3.75) suitable for acidic soils.

### RNA extraction and sequencing

Total RNA was extracted from panicle and root samples using a Takara MiniBEST RNA extraction kit (CAS No 9767, Japan). Six sample replicates were used for the organs and time points. DNase treatment was performed to remove DNA contamination. Library preparation was performed according to the manufacturer’s instructions using an mRNA purification kit (Library kit: TruSeq Stranded Total RNA LT Sample Prep Kit (Plant); protocol: TruSeq Stranded Total RNA Sample Prep Guide, Part #15031048 Rev.E; Reagent: TruSeq 3000 4000 SBS Kit v3). RNA fragments were randomly selected and purified for short-read sequencing, after which the fragmented RNA was reverse transcribed to generate cDNA. Adapters were attached to both ends of the cDNA fragments, and PCR amplification was performed. Fragments with insert sizes of 200–400 bp were selected for paired-end sequencing. Sequencing was conducted on a HiSeq 4000 System (Illumina), operated using HCS 3.3.52 software (Illumina, Inc., 9885 Towne Centre Drive, San Diego, CA 92121, USA) according to the provided HiSeq 3000 4000 System User Guide (Document #15066496 v05, HCS 3.3.52).

Raw sequencing reads were assessed for quality using FastQC v0.11.9. Adapter trimming and quality filtering were performed with Trimmomatic v0.39. Clean reads were aligned to the *O. sativa* japonica reference genome (IRGSP-1.0) using HISAT2 v2.2.1 with default parameters. Aligned reads were quantified using featureCounts v2.0.1 to produce a gene-level count matrix based on annotated genes. Differential gene expression analysis was carried out using the DESeq2 package (v1.34.0) in R. Raw gene counts were used as input, and genes with low overall expression (total count <10 across all samples) were pre-filtered. DESeq2’s median-of-ratios method was used for normalization to account for differences in library size. Differentially expressed genes (DEGs) were defined as those with an absolute log₂ (fold change) > 1. Gene Ontology (GO) and KEGG pathway enrichment analyses were performed to identify overrepresented biological functions and pathways among the DEGs.

## Results

### The *gs3* allele and low nitrogen reduce methane emissions synergistically by restructuring rhizosphere microbiota

Field trials revealed a significant genotype-by-environment interaction where the.

Milyang360 harboring the loss-of-function *gs3* allele demonstrated both superior agronomic stability and reduced methane emissions under low-nitrogen (LN) conditions compared to its parental line, Saeilmi. While LN conditions reduced plant vigor in both varieties, the yield loss in Milyang360 was only 7%, compared to 14% in Saeilmi (Supplementary Fig. 1). This agronomic resilience was coupled with a substantial environmental benefit, Milyang360 consistently emitted less methane throughout the growing season, with the LN-Milyang360 (combination of the *gs3* allele and LN treatment) resulting in a synergistic 42% reduction in cumulative emissions after the heading stage compared to the NN-Saeilmi control (Supplementary Fig. 2A and B). This reduction in methane flux was directly linked to a fundamental restructuring of the root-associated microbial communities.

16S rRNA gene amplicon sequencing of the rhizosphere and endosphere microbiota revealed distinct shifts in community structure and diversity influenced by nitrogen availability after heading stage (Figs 2A–D, Supplementary Fig. 2C and D). Microbial diversity, quantified through alpha diversity metrics such as ASV richness index, was consistently higher in the rhizosphere samples than in the endosphere samples, reflecting a more complex and heterogeneous soil environment (Fig. 2A). Rhizosphere communities were significantly more diverse under LN conditions.

To further investigate differences in community composition (beta diversity), we performed a principal coordinate analysis (PCoA). The PCoA plots, based on Bray–Curtis dissimilarity, showed a separation of bacterial communities between the rhizosphere and endosphere compartments (Supplementary Fig. 2C). Within each compartment, both the rice variety and nitrogen level significantly influenced the microbial community structure, with samples clustering distinctly according to these factors after the heading stage. In contrast, only nitrogen level influenced microbial community in tillering stage (Supplementary Fig. 2D).

The abundances of methanogenic and methanotrophic microbial groups were assessed using quantitative PCR (qPCR) targeting the *mcrA* gene of specific methanogenic groups, including *Methanosaetaceae* (MST), *Methanosarcinaceae* (MSC), *Methanobacteriales* (MBT), *Methanomicrobiales* (MMB), and *Methanocella*-specific (Met) [26, 27]. The results showed that methanogenic archaea were significantly more abundant in the NN and Saeilmi plots after the heading stage (Supplementary Fig. 3).

The cumulative abundances of methanogenesis related microorganisms decreased, whereas those of methanotrophic and nitrogen-fixing microorganisms increased in response to the *gs3* allele and low nitrogen conditions (Fig. 2B–D, Supplementary Table 1). Analysis of cumulative ASVs revealed no significant differences within the *gs3* (Fig. 2B), suggesting that the *gs3* allele does not broaden the diversity of methane-related microorganisms, but rather promotes the dominance of certain microbial taxa in the rhizosphere and endosphere.

A multilevel differential abundance analysis was conducted to dissect these microbial community dynamics, clearly separating the effect of the plant genotype (G) (the *gs3* allele) from the combined genotype-by-environment (GxE) interaction (Fig. 2C and D). First, we isolated the genotype effect by comparing NN-Milyang360 vs. NN-Saeilmi (Fig. 2C and D). This revealed a foundational shift driven by the *gs3* allele alone. In the rhizosphere (Fig. 2C), the *gs3* allele (NN-Milyang360) was associated with an increased abundance of *Nitrobacteraceae, Burkholderiaceae, Beijerinkiaceae, Methylococcaceae*, and *Methylothermaceae*. Conversely, the *GS3* allele (NN-Saeilmi) was linked to higher levels of *Clostridiaceae, Lachnospiraceae, Methanobacteriaceae, Methanosarcinaceae*, and *Streptococcaceae*. In the endosphere (Fig. 2D), the *gs3* allele similarly promoted a higher abundance of *Nitrobacteraceae, Burkholderiaceae, Beijerinckiaceae, Rhizobiaceae, Methylocystaceae, Methylococcaceae, Methylothermaceae*, and *Methylomonadaceae*, while the *GS3* allele was associated with *Acetobacteraceae* and *Lachnospiraceae*.

To evaluate the combined synergistic effect, we compared LN-Milyang360 vs. NN-Saeilmi (Fig. 2C and D). This comparison revealed that while the same families were suppressed or enriched, the magnitude of this difference was dramatically amplified.

### Metagenome-resolved analysis reveals distinct functional guilds driving methane dynamics

To link specific microbial lineages to ecosystem functions, we reconstructed 52 high-quality MAGs from the rhizosphere metagenome data, all with >60% completeness and <10% contamination (Fig. 3). Specifically, the presence of the *gs3* allele was associated with an increased abundance of MAGs related to methanotrophy and nitrogen metabolism, MAG04 and MAG24 (*Methylococcaceae*), MAG16 and MAG26 (*Beijerinckiaceae*), and MAG02 and MAG22 (*Nitrobacteraceae*). In contrast, the *Gs3* allele also promoted the enrichment of methanogenesis-associated MAGs, including MAG34 (*Syntrophobacteraceae*), MAG15 (*Oscillospiraceae*), MAG51 (*Lachnospiraceae*), MAG49 and MAG52 (*Streptococcaceae*), as well as MAG08 and MAG47 (*Methanosarcinaceae*).

**Figure 4:**
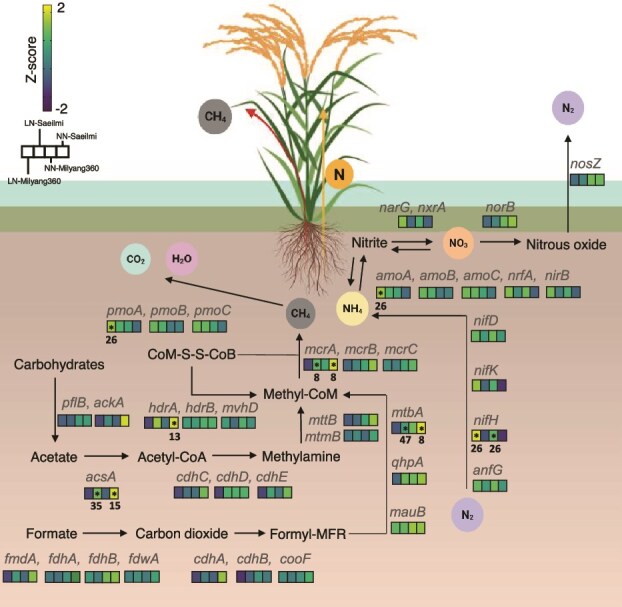
Schematic metabolic pathways for methane and nitrogen transformations mediated by rice rhizosphere microbial communities under different genotype and nitrogen condition. Microbial nitrogen and methane metabolic pathways reconstructed from metagenomic data in the rice rhizosphere under different genotype and nitrogen fertilization treatments. Genes were annotated using KEGG orthologs, and their relative abundances are represented as Z-scores. Methanogenesis pathways include formate oxidation (*fmdA, fdhA, fdhB, fdwA*), fermentation pathway (*cdhA, cdhB, cdhC, cdhD, cdhE, cooF*), and acetate conversion (*pflB, ackA, acsA*). Methyl-CoM formation and reduction are mediated *by mcrABC, hdrAB, mvhD*, and *mta, mtb, mtm* gene clusters. Methane oxidation is indicated by the presence of *pmoABC* (particulate methane monooxygenase). Nitrogen cycling genes include *nifHDK, anfG* (nitrogen fixation), *amoABC, nxrA* (nitrification), *narG, nosZ, norB* (denitrification), *nifA*, and *nirB* (nitrate reduction to ammonium). An asterisk (*) indicates that a specific MAG contributes over 15% of the community’s total gene abundance. Numbers marked under asterisk are annotated MAGs.

This high-emission condition was characterized by the enrichment of a syntrophic methanogenic consortium, including key methanogens (e.g. MAG08 *Methanosacrcinaceae*, MAG13, MAG33 *Methanobacteriaceae*) and their associated fermentative and syntrophic partners (e.g. MAG34 *Syntrophobacteraceae*, MAG15 *Oscillospiraceae*, MAG49 *Streptococcaceae*).Comprehensive genomic analysis revealed a complete repertoire of genes required for methanogenesis, including the key enzyme methyl-coenzyme M reductase (*mcrABC*) and pathways for both hydrogenotrophic and methylotrophic methane production (Fig. 4). The aggregated community-level metagenome further reinforced this, showing the highest abundance of genes for formylmethanofuran dehydrogenase (*fmdA*), carbon monoxide dehydrogenase (*cdhCDE*), acetate conversion (*acsA, ackA*), heterodisulfide reductase (*hdrA*), and the final methanogenic step (*mcrA*) in the NN treatments, particularly NN-Saeilmi (Fig. 3 and 4). MAG13, MAG15, MAG8 were dominant contributor of NN-Saeilmi community contributing over 15% of total gene abundance to *hdrA, acsA, mtbA*, and *mcrA* (Fig. 3 and 4).

A functionally opposite microbial guild was assembled in the rhizosphere of the Milyang360 genotype under low nitrogen. This low-emission environment was characterized by a decreased abundance of methanogenesis-related enzymes, accompanied by an increased presence of microbial communities associated with methane oxidation and nitrogen fixation. This functional shift was driven by the synergistic enrichment of methanotrophic MAGs, most MAG04 (*Methylococcaceae*) and MAG16, MAG26 (*Beijerinckiaceae*), MAG02, MAG11 (*Nitrobacteraceae*), which were highly abundant in the LN-Milyang360 treatment (Fig. 3). Among these, the genomes of MAG26, MAG16, and MAG04 contained the complete particulate methane monooxygenase operon (*pmoABC*), which serves as the canonical genetic marker for methane oxidation. Additionally, the genomes of MAG26, MAG16, MAG04, MAG02, and MAG11 possessed the nitrogenase gene *nifH*. In metagenomic datasets, particulate methane monooxygenase (*pmo*) genes are often co-annotated with ammonia monooxygenase (*amo*) genes due to their evolutionary homology, high amino acid sequence similarity, and shared conserved motifs [28, 29]. The entire community reflected this specialization, with the aggregated abundance of *pmoABC, amoABC, nifH, narG* genes being highest in the LN-Milyang360 treatment, and MAG26 identified as the dominant contributor contributing over 15% of total gene abundance of *nifH, amoA*, and *pmoA* (Fig. 4).

The community-level metabolic potential, reconstructed from the entire metagenome, corroborated these MAG-centric findings (Fig. 3 and 4). The aggregated abundance of genes involved in all major methanogenesis pathways including formate oxidation (*fdhA, B*), acetate conversion (*acsA, ackA*), and the final reduction to methane (*mcrA*) was high in the Saeilmi and more in NN-Saeilmi treatments. In contrast, the aggregated abundance of methane oxidation genes (*pmoA*) and nitrogen fixation (*nifH*) was highest in the LN-Milyang360 community (Fig. 3 and 4). This functional dichotomy, evident at both the individual genome and whole-community levels, provides strong evidence that the shift in the balance between methanogenesis and methanotrophy is the primary driver of the observed differences in methane emissions.

### Interaction analysis of methane and nitrogen metabolisms related functional gene

Genotype-by-environment (*gs3* × Nitrogen) interaction effect observed for the core genes of methanogenesis. This was most prominent for genes in the final methane production step (*mcrA, P* = .035), acetate conversion pathways (*ackA, P* = .025; *acsA, P* = .040), and associated heterodisulfide reductase (*hdrA, P* = .045). This powerful interaction signifies that the *gs3* allele’s effect on the methanogenic community is not constant but is dramatically dependent on nitrogen availability, with the NN-Saeilmi condition consistently showing the highest potential for methanogenesis (Supplementary Table 2).

Several key microbial functions were predominantly controlled by the plant’s genotype, regardless of the nitrogen level. This was most evident for nitrogen fixation genes, where *nifH* (*P* = .001), *nifD* (*P* = .030), *nifK* (*P* = .020), and *anfG* (*P* = .025) all showed a significant difference driven by the *gs3* allele alone. Similarly, several genes involved in nitrification/denitrification (*amoC, P* = .042; *nrfA, P* = .017; *nirB, P* = .022; *narG, P* = .018), and the acetyl-CoA pathway (*cdhE, P* = .045) were also primarily regulated by genotype. This suggests the *gs3* allele has a direct and robust influence on selecting for these specific microbial guilds (Supplementary Table 1).

### Transcriptome profiling of the *gs3* allele after LN treatment

**Figure 5:**
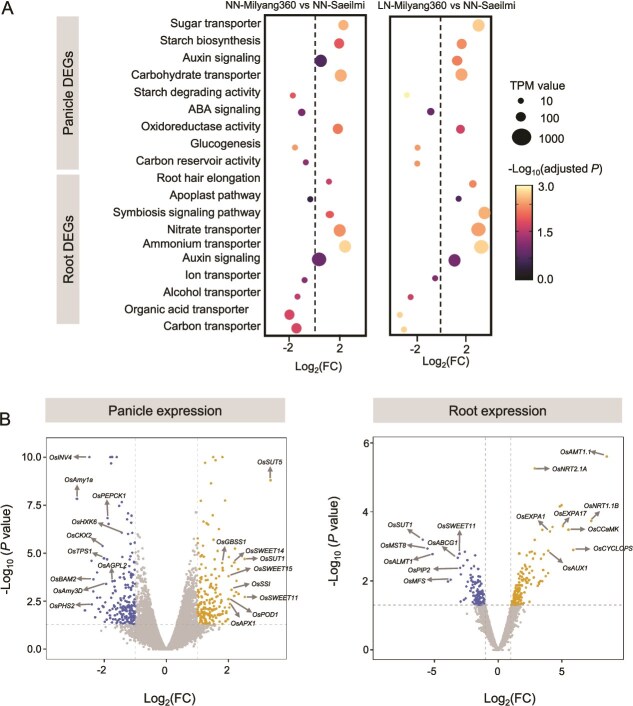
Differential gene expression in panicles and roots of rice under different nitrogen conditions and genotypes. (A) Bubble plots showing enriched functional categories of differentially expressed genes (DEGs) in panicles and roots between Milyang360 and Saeilmi varieties under NN and LN conditions. The x-axis represents log₂ fold change (Log₂FC), bubble size indicates transcript abundance (TPM), and bubble color corresponds to the –log₁₀(adjusted *P*) value. (B) Volcano plot of DEGs in panicles and roots that compared between LN-Milyang360 and NN-Saeilmi. The x-axis shows Log₂FC and the y-axis shows log₁₀(*P*) value. Significantly downregulated genes are positioned on the left (e.g., blue dots), while significantly upregulated genes are on the right (e.g., yellow dots); center-positioned dots (e.g., gray) represent no significant changes. Selected rice genes are labeled.

To elucidate the molecular mechanisms by which the *gs3* loss-of-function allele modulates plant physiology and rhizosphere interactions, we conducted a comprehensive transcriptome analysis of panicle and root tissues. Differential gene expression (DEG) analysis, visualized through volcano plots (Fig. 5B), and subsequent functional enrichment analysis (Fig. 5A) revealed distinct transcriptional reprogramming in Milyang360 compared to Saeilmi, particularly under low nitrogen (LN) conditions.

In the panicle tissues of Milyang360, we observed a significant upregulation of genes associated with carbon transport and oxidoreductase activity. Specifically, key rice sugar transporter genes such as *OsSUT1, OsSUT5*, and members of the *OsSWEET* family were significantly upregulated that exporting carbon from the plant’s roots into the rhizosphere [30]. Conversely, genes related to carbon reservoir activity, glucogenesis, and starch degrading activity were down regulated.

More profound transcriptional changes were observed in the root tissues, providing a direct link to the altered rhizosphere microbiome. The expression of genes encoding carbon and organic acid transporters was significantly suppressed in Milyang360 roots. For instance, the expression of the sucrose transporter *OsSUT1* and the aluminum-activated malate transporter *OsALMT1* was reduced (Fig. 5A and B, Supplementary Fig. 4, Supplementary Table 6). This downregulation directly limits the efflux of carbon substrates into the rhizosphere, thereby starving the methanogenic archaea that depend on these root exudates. In contrast, genes related to symbiotic signaling pathways and root hair elongation genes (*OsAUX1, OsEXPA17*) showed significant upregulation in Milyang360 roots (Fig. 3A and B, Supplementary Fig. 5, Supplementary Table 5). Key rice genes in plant- microorganism symbiosis, including *OsCCaMK* and *OsCYCLOPS*, were highly upregulated DEGs (Fig. 5A and B, Supplementary Fig. 5, Supplementary Table 6). These transcriptomic findings were further validated by qRT-PCR analysis, which confirmed the expression patterns of key selected rice genes (Fig. 5A and B, Supplementary Fig. 4).

## Discussion

### The *gs3* allele reshapes plant physiology to limit substrates for methanogenesis

A key mechanistic finding of current study, distinct from previous study [9], is that the *gs3* allele’s effect extends plant transcriptome changes and modulation of the specific heterotroph in the rhizosphere beyond simple carbon allocation. Our transcriptomic analysis revealed a significant upregulation of genes related to root hair elongation (e.g. *OsAUX1* and *OsEXPA17*) and, critically, key plant- microorganism symbiosis pathways including *OsCCaMK* and *OsCYCLOPS*. This is a pivotal discovery, *OsCCaMK* is a master regulator for microbial symbiosis. Its upregulation suggests a potential role in facilitating the accommodation of beneficial microorganisms, rather than solely passively starving methanogens [8, 31, 32]. Moreover, LN-Milyang360 plants had significantly reduced expression of genes involved in carbon transporter (*OsSUT1* and *OsMST8*) and organic acid transporter (*OsALMT1*) compared to the NN-Saeilmi (Fig. 5A and B, Supplementary Fig. 4, Supplementary Table 6). This downregulation curtails the efflux of photosynthates and other organic compounds from the roots, reducing the release of carbon and organic acids. Our finding aligns with previous hypotheses that redirecting carbon allocation from roots to grains can be an effective strategy for methane mitigation. [27, 30, 33, 34]. This resource reallocation inherently limits the carbon available for root exudation, which essentially starves methane-producing microorganisms (methanogens) in the soil that depend on these substances, leading to lower methane emissions. [8–10, 35]. Concurrently, genes related to starch biosynthesis (*OsSSI* and *OsGBSS1*), carbon transporter (*OsSUT1, OsSUT5, OsSWEET11*, and *OsMST1*), and oxidoreductase activity (*OsAPX1* and *OsPOD1*) were upregulated in the panicles of Milyang360, while genes involved in starch degradation (*OsAmy1A* and *OsBAM2*), gluconeogenesis (*OsINV4, OsPEPCK1*, and *OsHXK6*), and carbon reservoir activity (*OsAGPL2* and *OsTPS1*) were downregulated (Fig. 5A and B, Supplementary Fig. 4, Supplementary Table 6). These results indicate that the *gs3* allele enhances carbon partitioning to the panicle for grain filling, which is consistent with its known function in increasing grain size [36–40]. This resource reallocation inherently limits the carbon available for root exudation, a conclusion supported by a previous study showing that Milyang360 secretes fewer carbon-derived substances including organic acids and carbohydrates [9, 10, 34]. Nitrogen uptake transporters (*OsNRT1.1B* and *OsNRT2.1A*) were upregulated in the Milyang360 genotype (Fig. 5A and B, Supplementary Fig. 4, Supplementary Table 6). This upregulation led to increased protein content in the grain (Supplementary Fig. 1). In addition, enhanced root hair development can create more oxic zones around the roots [35, 41]. These zones promote methanotroph inhabitation and may help with methane reduction with nitrogen uptake.

### Microbial community restructuring shift from methane producers to consumers and nitrogen fixer

Our results demonstrate that the *gs3* allele does not merely suppress methanogens, but fosters a synergistic enrichment of beneficial microorganisms geared towards methane consumption (methanotrophs) and nitrogen cycling (nitrogen-fixers).

The LN-Milyang360 treatment fostered a community geared towards methane consumption and nitrogen cycling. We observed a significant increase in the abundance of methanotrophs, particularly methanotrophs from families like *Methylocystaceae, Methylococcaceae*, and *Beijerinckiaceae* (Fig. 2C and D). These bacteria are potent methane oxidizers [42–47], and their enrichment enables the simultaneous oxidation of methane and fixation of atmospheric nitrogen in the soil [48–56]. An abundance of methanotrophs in the rice root environment inhibits rice yield loss and reduces methane emissions under low-nitrogen conditions [31, 32]. The upregulation of plant symbiosis pathway genes (*OsCCaMK, OsCYCLOPS*) in Milyang360 roots (Fig. 5B, Supplementary Fig. 4) likely facilitates the colonization of these beneficial microorganisms in rhizosphere and endosphere [31, 57, 58]. Our MAG analysis identified MAG26 (*Beijerinckiaceae*) as a keystone microorganism orchestrating this functional shift, which is predominantly found in the of Milyang360 (Fig. 2C and D). This microorganism was not only highly enriched in the Milyang360 environment but, critically, its genome revealed a remarkable metabolic versatility. As a dominant contributor to the community-wide abundance of both the particulate methane monooxygenase (*pmoA*), ammonia monooxygenase (*amoA*), and nitrogenase (*nifH*) genes (Fig. 3 and 4). MAG26 embodies the elegant solution this ecosystem has found, a single organism that simultaneously mitigates greenhouse gas emissions by consuming methane and enhances plant nutrition by fixing atmospheric nitrogen [59–61]. Such dual-function microorganisms are especially advantageous under low-nitrogen input systems, as they compensate for reduced fertilizer application by biological nitrogen fixation while also limiting methane flux through oxidation. This coupling of carbon and nitrogen cycling within single microbial genomes represents a powerful ecological trait favored by the *gs3*-engineered root environment. This dual role is consistent with previous observations of methanotrophs like *Methylocystis* functioning as facultative diazotrophs [26, 62]. Their enrichment in Milyang360 plots reinforces the idea that host plant genetics can shape not only the taxonomic makeup but also the metabolic architecture of the rhizosphere to achieve multifunctional benefits.

In the high-emission NN-Saeilmi environment, the microbiome was dominated by a syntrophic consortium of fermentative bacteria (*Clostridiaceae, Lachnospiraceae*) and methanogenic archaea (*Methanosarcinaceae, Methanobacteriaceae*) [63, 64] (Fig. 2C and D). Our MAG analysis provided direct evidence supporting this finding by identifying specific MAGs more abundant in the NN-Saeilmi environment that possess complete genetic pathways for methanogenesis, including the key gene *mcrA* (Fig. 3 and 4). At the community level, the high abundance of genes involved in acetate conversion (*ackA, acsA*) and heterodisulfide reductase (*hdrA*) further reinforces this functional specialization [65] (Fig. 3 and 4). The microbial consortia were dominated by methanogenic taxa including *Methanosarcinaceae* (MAG08), *Methanobacteriaceae* (MAG13), and fermenters such as *Oscillospiraceae* (MAG15). Functional gene analysis confirmed the presence of the full methanogenesis machinery, including *mcrA, fdhAB, acsA*, and *ackA*, which together facilitate carbon flux toward methane production under anoxic soil conditions (Fig. 4). These MAGs are observed in NN-Saeilmi, as they harbor functional genes accounting for over 15% of the total (Fig. 4). The community-level enrichment of these genes suggests not only a favorable niche for methanogenesis but also cooperative metabolic interactions between fermenters and methanogens a classical syntrophic consortium that amplifies methane flux. The enrichment of these keystone methanogens is central to the high methane flux observed in the Saeilmi rhizosphere, while their suppression in the *gs3* allele background is critical for reducing emissions.

### Synergistic effect of low nitrogen and the *gs3* allele

The *gs3* allele alone contributed to lower emissions, its effect was most pronounced under LN conditions, leading to a 42% reduction in cumulative methane emissions after the heading stage compared to NN-Saeilmi (Supplementary Fig. 2B). The two-way ANOVA confirmed this strong genotype-by-environment (G × N) interaction for the abundance of key methanogenesis genes like *mcrA* and *ackA* (Supplementary Table 2). Furthermore, the abundances of genes related to methanogenesis, methanotrophy, and nitrogen metabolism were significantly affected by the *gs3* allele, and most of these genes showed a synergistic effect with the nitrogen level (Supplementary Table 2). These included key genes such as *pmoAC, mcrAB, ackA, hdrA, mttB, mtbA, acsA, cdhE, fmdA, cdhA, anfG, nifHKD, amoABC, nrfA, nirB, narG*, and *nxrA*. This suggests that the low nitrogen environment enhances the plant’s strategy of restricting carbon flow to the rhizosphere, thereby amplifying the competitive advantage of methanotrophs over methanogens.

The LN-Milyang360 condition promoted a community enriched in nitrogen-fixing bacteria, including *Nitrobacteraceae, Nitromonadaceae*, and *Burkholderiaceae* (Fig. 2C and D). The increased abundance of nitrogenase genes (*nifHDK*) at both the community (Fig. 4) and individual MAG level (e.g. MAG26, Fig. 3) indicates that these microorganisms are fixing atmospheric nitrogen [66–69]. This biological nitrogen fixation likely compensates for the reduced fertilizer input, a conclusion strongly supported by the agronomic traits observed in Milyang360. Specifically, this variety exhibited superior yield stability (only 7% loss vs. 14% in Saeilmi) and maintained consistent grain protein content under low nitrogen conditions (Supplementary Fig. 1). The *gs3* quantitative trait locus (QTL), which encodes a G-protein γ-subunit involved in the upstream regulation of nitrogen metabolism [11, 12, 70], plays a pivotal role in this process. The *gs3* allele offers a dual advantage, it not only helps mitigate methane emissions but also enhances nitrogen use efficiency (NUE). These combined benefits make *gs3* a particularly promising target for advancing sustainable agricultural practices [71, 72].

Our data show that the low-nitrogen treatment suppresses strictly anaerobic methanogenic taxa while enriching aerobic/microaerophilic methanotrophs and nitrogen-fixers. To confirm the exact mechanism driving this significant anaerobic-to-aerobic guild shift, future studies would be required. This mechanism, which appears to be synergistic with the carbon limitation mechanism, likely involves unmeasured physicochemical factors. Future investigations should therefore focus on quantifying soil redox potential and root radial oxygen loss under different nitrogen regimes, as these factors are known to be dominant controls that structure anoxic and oxic niches in the rhizosphere.

By selecting for genetic traits like the *gs3* allele, it is possible to develop new rice cultivars that inherently support a microbiome that minimizes greenhouse gas emissions. The synergistic effect with reduced nitrogen fertilization is particularly noteworthy, offering a pathway to decrease both methane emissions and the economic and environmental costs associated with heavy fertilizer use. The *gs3* allele, therefore, represents a valuable genetic resource for breeding high-yield, low-emission rice varieties that are better adapted to low-input farming systems, contributing to a more sustainable and climate-resilient future for global rice production.

## Conclusion

Milyang360 contains the *gs3* loss-of-function allele and can be used as a model to understand the influence of genetic traits on microbial community dynamics and greenhouse gas emissions. The loss-of-function *gs3* allele plays a dual role in reducing methane emissions and enhancing nitrogen-use efficiency by promoting methane-oxidizing and nitrogen-fixing microorganisms in both the rhizosphere and endosphere. It has a synergistic effect with reduced nitrogen fertilization. These results align with the principles of sustainable agriculture, which aim to reduce methane emissions and fertilizer use.

## Supplementary Material

wraf284_Supplemental_Figures_Table

## Data Availability

The sequence data were deposited in the NCBI Sequence Read Archive database under NCBI BioProjects PRJNA1244931 and PRJNA1346890.

## References

[1] Bridgham SD, Cadillo-Quiroz H, Keller JK. et al. Methane emissions from wetlands: biogeochemical, microbial, and modeling perspectives from local to global scales. *Glob Chang Biol* 2013;19:1325–46. 23505021 10.1111/gcb.12131

[2] Liu Y, Whitman WB. Metabolic, phylogenetic, and ecological diversity of the methanogenic archaea. *Ann N Y Acad Sci* 2008;1125:171–89. 10.1196/annals.1419.019 18378594

[3] Peng S, Huang J, Sheehy JE. et al. Rice yields decline with higher night temperature from global warming. *Proc Natl Acad Sci USA* 2004;101:9971–5. 15226500 10.1073/pnas.0403720101 PMC454199

[4] Conrad R . Microbial ecology of methanogens and methanotrophs. *Adv Agron* 2007;96:1–63.

[5] Conrad R . Control of microbial methane production in wetland rice fields. *Nutr Cycl Agroecosyst* 2002;64:59–69.

[6] Ettwig KF, Butler MK, Le Paslier D. et al. Nitrite-driven anaerobic methane oxidation by oxygenic bacteria. *Nature* 2010;464:543–8. 10.1038/nature08883 20336137

[7] Lieberman RL, Rosenzweig AC. Biological methane oxidation: regulation, biochemistry, and active site structure of particulate methane monooxygenase. *Crit Rev Biochem Mol Biol* 2004;39:147–64. 15596549 10.1080/10409230490475507

[8] Kwon Y, Jin Y, Lee J-H. et al. Rice rhizobiome engineering for climate change mitigation. *Trends Plant Sci* 2024;29:1299–309. 10.1016/j.tplants.2024.06.006 39019767

[9] Kwon Y, Lee J-Y, Choi J. et al. Loss-of-function gs3 allele decreases methane emissions and increases grain yield in rice. *Nat Clim Chang* 2023;13:1329–33.

[10] Su J, Hu C, Yan X. et al. Expression of barley susiba2 transcription factor yields high-starch low-methane rice. *Nature* 2015;523:602–6. 10.1038/nature14673 26200336

[11] Mao H, Sun S, Yao J. et al. Linking differential domain functions of the gs3 protein to natural variation of grain size in rice. *Proc Natl Acad Sci USA* 2010;107:19579–84. 10.1073/pnas.1014419107 20974950 PMC2984220

[12] Fan C, Xing Y, Mao H. et al. Gs3, a major qtl for grain length and weight and minor qtl for grain width and thickness in rice, encodes a putative transmembrane protein. *Theor Appl Genet* 2006;112:1164–71. 16453132 10.1007/s00122-006-0218-1

[13] Kim GW, Jeong ST, Kim PJ. et al. Influence of nitrogen fertilization on the net ecosystem carbon budget in a temperate mono-rice paddy. *Geoderma* 2017;306:58–66.

[14] Kim GW, Gutierrez-Suson J, Kim PJ. Optimum n rate for grain yield coincides with minimum greenhouse gas intensity in flooded rice fields. *Field Crop Res* 2019;237:23–31.

[15] Banger K, Tian H, Lu C. Do nitrogen fertilizers stimulate or inhibit methane emissions from rice fields? *Glob Chang Biol* 2012;18:3259–67. 28741830 10.1111/j.1365-2486.2012.02762.x

[16] Schimel J . Rice, microbes and methane. *Nature* 2000;403:375–7. 10667774 10.1038/35000325

[17] Simmons T, Caddell DF, Deng S. et al. Exploring the root microbiome: extracting bacterial community data from the soil, rhizosphere, and root endosphere. *J Vis Exp* 2018;135:57561. 10.3791/57561 PMC6101100 29782021

[18] Louca S, Parfrey LW, Doebeli M. Decoupling function and taxonomy in the global ocean microbiome. *Science* 2016;353:1272–7. 10.1126/science.aaf4507 27634532

[19] Bolger AM, Lohse M, Usadel B. Trimmomatic: a flexible trimmer for illumina sequence data. *Bioinformatics* 2014;30:2114–20. 10.1093/bioinformatics/btu170 24695404 PMC4103590

[20] Li D, Liu C-M, Luo R. et al. Megahit: an ultra-fast single-node solution for large and complex metagenomics assembly via succinct de bruijn graph. *Bioinformatics* 2015;31:1674–6. 10.1093/bioinformatics/btv033 25609793

[21] Hyatt D, Chen G-L, LoCascio PF. et al. Prodigal: prokaryotic gene recognition and translation initiation site identification. *BMC Bioinformatics* 2010;11:119. 20211023 10.1186/1471-2105-11-119 PMC2848648

[22] Buchfink B, Xie C, Huson DH. Fast and sensitive protein alignment using diamond. *Nat Methods* 2015;12:59–60. 10.1038/nmeth.3176 25402007

[23] Abdallah RZ, Elbehery AH, Ahmed SF. et al. Deciphering the functional and structural complexity of the solar lake flat mat microbial benthic communities. *Msystems* 2024;9:e00095-24. 38727215 10.1128/msystems.00095-24 PMC11237645

[24] Cantalapiedra CP, Hernández-Plaza A, Letunic I. et al. Eggnog-mapper v2: functional annotation, orthology assignments, and domain prediction at the metagenomic scale. *Mol Biol Evol* 2021;38:5825–9. 10.1093/molbev/msab293 34597405 PMC8662613

[25] Potter SC, Luciani A, Eddy SR. et al. Hmmer web server: 2018 update. *Nucleic Acids Res* 2018;46:W200–4. 10.1093/nar/gky448 29905871 PMC6030962

[26] Mäkipää R, Leppänen SM, Munoz SS. et al. Methanotrophs are core members of the diazotroph community in decaying Norway spruce logs. *Soil Biol Biochem* 2018;120:230–2.

[27] Jin Y, Liu T, Hu J. et al. Reducing methane emissions by developing low-fumarate high-ethanol eco-friendly rice. *Mol Plant* 2025;18:333–49. 10.1016/j.molp.2025.01.008 39904305

[28] Cupples AM, Dang H, Foss K. et al. An investigation of soil and groundwater metagenomes for genes encoding soluble and particulate methane monooxygenase, toluene-4-monoxygenase, propane monooxygenase and phenol hydroxylase. *Arch Microbiol* 2024;206:363. 39073473 10.1007/s00203-024-04088-z

[29] Holmes AJ, Costello A, Lidstrom ME. et al. Evidence that participate methane monooxygenase and ammonia monooxygenase may be evolutionarily related. *FEMS Microbiol Lett* 1995;132:203–8. 7590173 10.1016/0378-1097(95)00311-r

[30] Sasse J, Martinoia E, Northen T. Feed your friends: do plant exudates shape the root microbiome? *Trends Plant Sci* 2018;23:25–41. 10.1016/j.tplants.2017.09.003 29050989

[31] Bao Z, Watanabe A, Sasaki K. et al. A rice gene for microbial symbiosis, oryza sativa ccamk, reduces ch4 flux in a paddy field with low nitrogen input. *Appl Environ Microbiol* 2014;80:1995–2003. 10.1128/AEM.03646-13 24441161 PMC3957643

[32] Minamisawa K, Imaizumi-Anraku H, Bao Z. et al. Are symbiotic methanotrophs key microbes for n acquisition in paddy rice root? *Microbes Environ* 2016;31:4–10. 10.1264/jsme2.ME15180 26960961 PMC4791114

[33] Hu J, Bedada G, Sun C. et al. Fumarate reductase drives methane emissions in the genus oryza through differential regulation of the rhizospheric ecosystem. *Environ Int* 2024;190:108913. 39079335 10.1016/j.envint.2024.108913

[34] Hu J, Bettembourg M, Xue L. et al. A low-methane rice with high-yield potential realized via optimized carbon partitioning. *Sci Total Environ* 2024;920:170980. 38373456 10.1016/j.scitotenv.2024.170980

[35] Chen Y, Zhang Y, Li S. et al. Osrga1 optimizes photosynthate allocation for roots to reduce methane emissions and improve yield in paddy ecosystems. *Soil Biol Biochem* 2021;160:108344.

[36] Xu X, Zhiguo E, Zhang D. et al. Osyuc11-mediated auxin biosynthesis is essential for endosperm development of rice. *Plant Physiol* 2021;185:934–50. 10.1093/plphys/kiaa057 33793908 PMC8133553

[37] Song Y, Jiang Y, Chen F. et al. Nitrogen optimization enhances grain filling and starch biosynthesis in japonica rice: physiological regulation of carbon–nitrogen metabolism and synthase activities. *Cereal Res Commun* 2025;53:2505–2516.

[38] Liu D, Li M-j, Luo J-s. et al. Overexpression of osstp1 increases grain yield via enhancing carbohydrate metabolism and transport in rice. *Planta* 2025;261:5. 10.1007/s00425-024-04579-9 39623007

[39] Dong N, Jiao G, Cao R. et al. Oslesv and osesv1 promote transitory and storage starch biosynthesis to determine rice grain quality and yield. *Plant Commun* 2024;5:100780. 38581128 10.1016/j.xplc.2024.100893 PMC11287174

[40] Mathan J, Singh A, Ranjan A. Sucrose transport and metabolism control carbon partitioning between stem and grain in rice. *J Exp Bot* 2021;72:4355–72. 10.1093/jxb/erab066 33587747

[41] Gilroy S, Jones DL. Through form to function: root hair development and nutrient uptake. *Trends Plant Sci* 2000;5:56–60. 10664614 10.1016/s1360-1385(99)01551-4

[42] Bowman J . The methanotrophs—the families methylococcaceae and methylocystaceae. *The Prokaryotes* 2006;5:266–89.

[43] Hanson RS, Hanson TE. Methanotrophic bacteria. *Microbiol Rev* 1996;60:439–71. 8801441 10.1128/mr.60.2.439-471.1996 PMC239451

[44] Sakoda M, Tokida T, Sakai Y. et al. Mitigation of paddy field soil methane emissions by betaproteobacterium azoarcus inoculation of rice seeds. *Microbes Environ* 2022;37:ME22052. 36517028 10.1264/jsme2.ME22052 PMC9763044

[45] Whittenbury R, Davies SL, Davey J. Exospores and cysts formed by methane-utilizing bacteria. *Microbiology* 1970;61:219–26. 10.1099/00221287-61-2-219 5476892

[46] Dilworth MJ, Eady RR, Eldridge ME. The vanadium nitrogenase of azotobacter chroococcum. Reduction of acetylene and ethylene to ethane. *Biochem J* 1988;249:745–51. 3162672 10.1042/bj2490745 PMC1148769

[47] Lobo AL, Zinder SH. Diazotrophy and nitrogenase activity in the archaebacterium methanosarcina barkeri 227. *Appl Environ Microbiol* 1988;54:1656–61. 16347675 10.1128/aem.54.7.1656-1661.1988 PMC202723

[48] Hakobyan A, Liesack W. Unexpected metabolic versatility among type ii methanotrophs in the alphaproteobacteria. *Biol Chem* 2020;401:1469–77. 10.1515/hsz-2020-0200 32769217

[49] Murrell JC, Dalton H. Nitrogen fixation in obligate methanotrophs. *Microbiology* 1983;129:3481–6.

[50] Shrestha M, Shrestha PM, Frenzel P. et al. Effect of nitrogen fertilization on methane oxidation, abundance, community structure, and gene expression of methanotrophs in the rice rhizosphere. *ISME J* 2010;4:1545–56. 10.1038/ismej.2010.89 20596069

[51] Graham DW, Chaudhary JA, Hanson RS. et al. Factors affecting competition between type i and type ii methanotrophs in two-organism, continuous-flow reactors. *Microb Ecol* 1993;25:1–17. 10.1007/BF00182126 24189703

[52] Amaral JA, Knowles R. Growth of methanotrophs in methane and oxygen counter gradients. *FEMS Microbiol Lett* 1995;126:215–20.

[53] Davamani V, Parameswari E, Arulmani S. Mitigation of methane gas emissions in flooded paddy soil through the utilization of methanotrophs. *Sci Total Environ* 2020;726:138570. 32305766 10.1016/j.scitotenv.2020.138570

[54] Belova SE, Baani M, Suzina NE. et al. Acetate utilization as a survival strategy of peat-inhabiting methylocystis spp. *Environ Microbiol Rep* 2011;3:36–46. 10.1111/j.1758-2229.2010.00180.x 23761229

[55] Holmes AJ, Roslev P, McDonald IR. et al. Characterization of methanotrophic bacterial populations in soils showing atmospheric methane uptake. *Appl Environ Microbiol* 1999;65:3312–8. 10427012 10.1128/aem.65.8.3312-3318.1999 PMC91497

[56] Oremland RS, Culbertson CW. Importance of methane-oxidizing bacteria in the methane budget as revealed by the use of a specific inhibitor. *Nature* 1992;356:421–3.

[57] Ikeda S, Okubo T, Takeda N. et al. The genotype of the calcium/calmodulin-dependent protein kinase gene (*ccamk*) determines bacterial community diversity in rice roots under paddy and upland field conditions. *Appl Environ Microbiol* 2011;77:4399–405. 10.1128/AEM.00315-11 21551283 PMC3127688

[58] Yano K, Yoshida S, Müller J. et al. Cyclops, a mediator of symbiotic intracellular accommodation. *Proc Natl Acad Sci USA* 2008;105:20540–5. 10.1073/pnas.0806858105 19074278 PMC2629324

[59] Tamas I, Smirnova AV, He Z. et al. The (d) evolution of methanotrophy in the beijerinckiaceae—a comparative genomics analysis. *ISME J* 2014;8:369–82. 10.1038/ismej.2013.145 23985741 PMC3906808

[60] Bay SK, Dong X, Bradley JA. et al. Trace gas oxidizers are widespread and active members of soil microbial communities. *Nat Microbiol* 2021;6:246–56. 10.1038/s41564-020-00811-w 33398096

[61] Gwak J-H, Awala SI, Nguyen N-L. et al. Sulfur and methane oxidation by a single microorganism. *Proc Natl Acad Sci USA* 2022;119:e2114799119. 35914169 10.1073/pnas.2114799119 PMC9371685

[62] Halsall DM, Gibson AH. Nitrogenase activity by diazotrophs grown on a range of agricultural plant residues. *Soil Biol Biochem* 1989;21:1037–43.

[63] Evans PN, Boyd JA, Leu AO. et al. An evolving view of methane metabolism in the archaea. *Nat Rev Microbiol* 2019;17:219–32. 10.1038/s41579-018-0136-7 30664670

[64] Wang S, Sun P, Liu J. et al. Distribution of methanogenic and methanotrophic consortia at soil-water interfaces in rice paddies across climate zones. *Iscience* 2023;26:107659. 36636345 10.1016/j.isci.2022.105851 PMC9829807

[65] Li D, Ni H, Jiao S. et al. Coexistence patterns of soil methanogens are closely tied to methane generation and community assembly in rice paddies. *Microbiome* 2021;9:1–13. 33482926 10.1186/s40168-020-00978-8 PMC7825242

[66] Estrada-De Los Santos P, Bustillos-Cristales R, Caballero-Mellado J. Burkholderia, a genus rich in plant-associated nitrogen fixers with wide environmental and geographic distribution. *Appl Environ Microbiol* 2001;67:2790–8. 11375196 10.1128/AEM.67.6.2790-2798.2001 PMC92940

[67] Watson SW, Valois FW, Waterbury JB. The family nitrobacteraceae. In: The Prokaryotes: A Handbook on Habitats, Isolation, and Identification of Bacteria. Springer, Berlin, Heidelberg, 1005–1022.

[68] Lücker S, Daims H. The family nitrospinaceae. In: The Prokaryotes. Springer, Berlin, Heidelberg, 231–237.

[69] Prosser JI, Head IM, Stein LY. The family nitrosomonadaceae. In: The Prokaryotes. Springer, Berlin, Heidelberg, 901–918.

[70] Liu Q, Wu K, Song W. et al. Improving crop nitrogen use efficiency toward sustainable green revolution. *Annu Rev Plant Biol* 2022;73:523–51. 10.1146/annurev-arplant-070121-015752 35595292

[71] Hawkesford MJ, Griffiths S. Exploiting genetic variation in nitrogen use efficiency for cereal crop improvement. *Curr Opin Plant Biol* 2019;49:35–42. 10.1016/j.pbi.2019.05.003 31176099 PMC6692496

[72] Yoon DK, Suganami M, Ishiyama K. et al. The gs3 allele from a large-grain rice cultivar, Akita 63, increases yield and improves nitrogen-use efficiency. *Plant Direct* 2022;6:e417. 35865075 10.1002/pld3.417 PMC9289216

