# Supplementary material for: Rice gs3 allele and low-nitrogen conditions enrich rhizosphere microbiota that mitigate methane emissions and promote beneficial crop traits
Source: ISME J. 2025 Dec 29;20(1):wraf284. doi: 10.1093/ismejo/wraf284 (PMC12815268; doi:10.1093/ismejo/wraf284)
Supplement: wraf284_Supplemental_Figures_Table [file wraf284_supplemental_figures_table.zip › Supplementary_materials_wraf284_figures.docx]

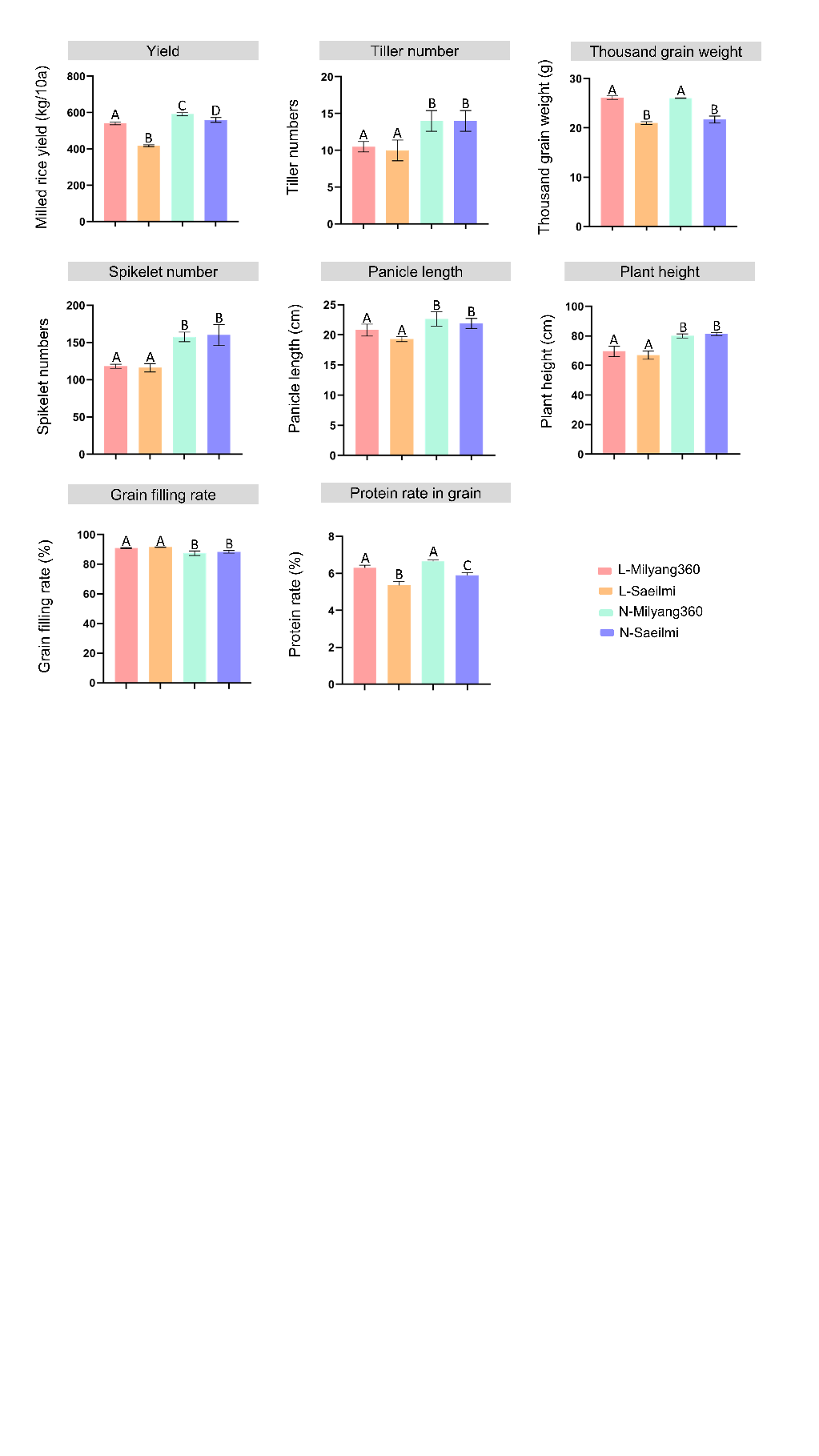


**Supplementary Fig. 1. Agronomic traits of Milyang360 and Saeilmi rice varieties under low and normal nitrogen conditions.**

Key agronomic traits of Milyang360 and Saeilmi rice varieties, including plant height, tiller number, panicle length, grain weight, and grain protein content across different nitrogen treatments (low-nitrogen and normal-nitrogen). Differences in plant morphology and development are highlighted, with different uppercase letters indicating statistical significance (*P* <0.05; Tukey’s HSD test). Error bars indicate the standard error.

**
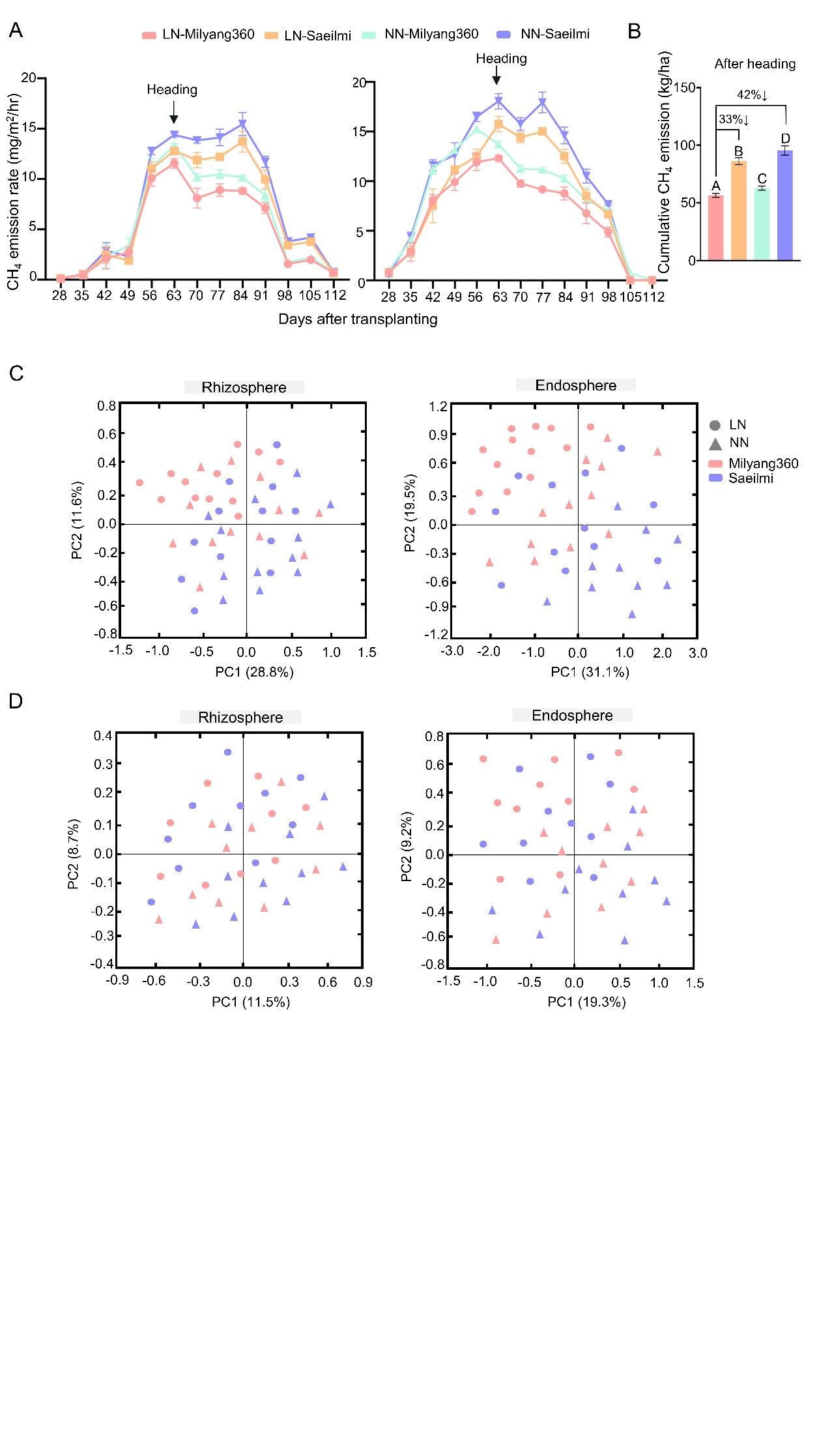
**

**Supplementary Fig. 2.** **Impacts of the *gs3* loss-of-function allele and nitrogen availability on methane emissions and microbial dynamics.**

(A) Temporal changes in the CH₄ emission rate (mg m⁻² hr⁻¹) from transplanting to maturity under LN and NN conditions for the Milyang360 and Saeilmi rice varieties in the Miryang (left) and Jinju (right) fields. The arrow indicates the heading stage. (B) Average cumulative methane emissions after the heading stage (two sites and two years). Error bars indicate the standard error. (C) Principal coordinate analysis (PCoA) plots based on Bray–Curtis dissimilarity showing the bacterial community structures in the rhizosphere (left) and endosphere (right) under different nitrogen levels and rice varieties in the after-heading and tillering stages. Symbols indicate nitrogen levels (circles, LN; triangles, NN), and colors indicate varieties. The percentages on the axes indicate the proportion of variation explained by each principal coordinate.


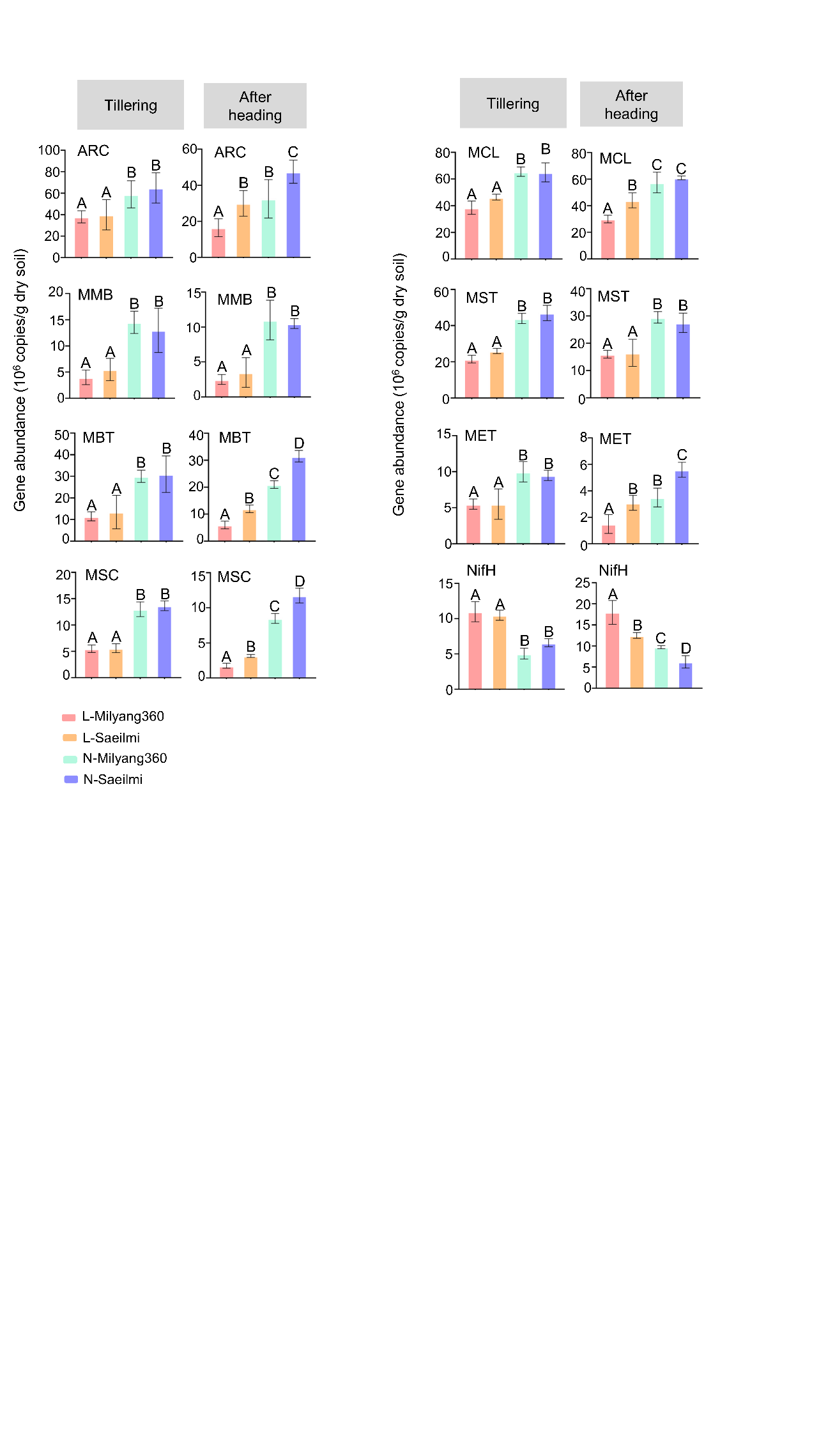


**Supplementary Fig. 3. Quantification of methanogen and nitrogen-fixing microbes using qPCR.**

Quantitative abundance of methanogenic families and N2-fixing related microbes using qRT-PCR with the rhizosphere and endosphere soil samples, measured at tillering and after heading stages under different nitrogen conditions (L-Milyang360, L-Saeilmi, N-Milyang360, N-Saeilmi), Quantification wasperformed for total archaea (ARC) and methanogens (MET) and the Methanosaetaceae (MST), Methanosarcinaceae (MSC), Methanobacteriales (MBT), Methanomicrobiales (MMB), and Methanocella-specific (Met), nitrogenase gene (NifH). Statistical differences between treatments are indicated by different upper-case letters (*P* <0.05; Tukey’s HSD test). Error bars indicate standard error.


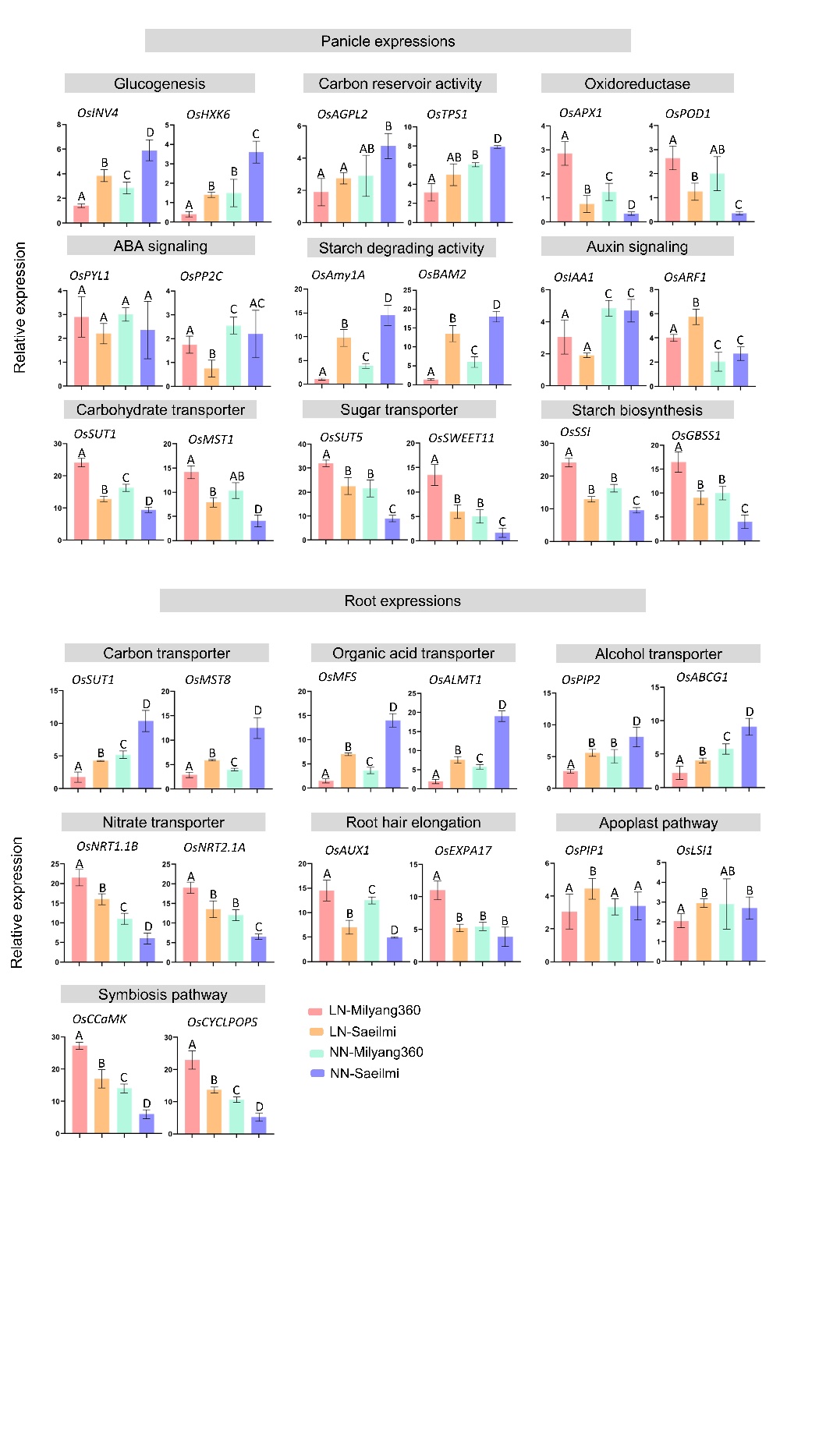


**Supplementary Fig. 4. RNA-seq validation of differentially expressed metabolic genes.**

Validation of the expression levels of key genes related to carbohydrate metabolism, organic acid transport, and symbiosis signaling in Milyang360 and Saeilmi rice varieties under low and normal nitrogen conditions. Validation was performed using quantitative real-time PCR (qRT-PCR), and the expression patterns were consistent with RNA-seq data. The bars represent the mean expression values, and the error bars indicate the standard deviation. Statistical differences between treatments and varieties are shown using different upper-case letters (*P* <0.05; Tukey’s HSD test). Error bars indicate the standard error.
