# Supplementary material for: Rice gs3 allele and low-nitrogen conditions enrich rhizosphere microbiota that mitigate methane emissions and promote beneficial crop traits
Source: ISME J. 2025 Dec 29;20(1):wraf284. doi: 10.1093/ismejo/wraf284 (PMC12815268; doi:10.1093/ismejo/wraf284)
Supplement: wraf284_Supplemental_Figures_Table [file wraf284_supplemental_figures_table.zip › Supplementary_materials_wraf284_table.docx]

| Function | Classification | Reference |
| --- | --- | --- |
| Methanogenesis | *Methanosarcinaceae, Methanobacteriaceae, Methanomicrobiaceae, Methanocellaceae,* | FAPROTAX [18] |
| Methanotrophy | *Methylococcaceae, Methylocystaceae, Methylacidiphilaceae, Beijerinckiaceae* |  |
| Nitrogen fixing | *Beijerinckiaceae, Rhizobiaceae, Nitrobacteraceae, Burkholderiaceae* |  |

**Supplementarty table 1.** Functional guild annotation of ASVs using FAPROTAX

**Supplementary table 2.** Two-way ANOVA p-values for the effects of factors on the abundances of functional gene, KEGG database entry numbers and descriptions.

| Functional gene | Factors (*P* -value) | | | | | | KEGG entry  number | Description |
| --- | --- | --- | --- | --- | --- | --- | --- | --- |
|  | Genotype (G) | | Nitrogen (N) | | G x N | |  |  |
|  | F value | P value | F value | P value | F value | P value |  |  |
| *pmoA* | 9.5 | 0.044 | 15.8 | 0.024 | 10.5 | 0.039 | K10944 | Particulate methane monooxygenase |
| *pmoB* | 0.43 | 0.550 | 21.1 | 0.009 | 0.06 | 0.818 | K10945 |  |
| *pmoC* | 8.64 | 0.042 | 10.45 | 0.032 | 2.27 | 0.206 | K10946 |  |
| *mcrA* | 35.67 | 0.012 | 30.11 | 0.013 | 42.56 | 0.035 | K00399 | Methyl-coenzyme M reductase subunit A |
| *mcrB* | 8.31 | 0.048 | 10.87 | 0.030 | 1.74 | 0.257 | K00400 |  |
| *mcrC* | 0.50 | 0.518 | 0.94 | 0.388 | 0.19 | 0.687 | K00401 |  |
| *pflB* | 0.61 | 0.480 | 10.58 | 0.038 | 0.39 | 0.566 | K00656 | Pyruvate formate-lyase |
| *ackA* | 38.24 | 0.009 | 44.12 | 0.008 | 76.47 | 0.025 | K00925 | Acetate kinase |
| *hdrA* | 35.00 | 0.010 | 52.33 | 0.025 | 33.33 | 0.045 | K03533 | Electron transfer proteins in methanogenesis |
| *hdrB* | 0.88 | 0.401 | 0.82 | 0.411 | 0.53 | 0.508 | K03534 |  |
| *mvhD* | 2.81 | 0.169 | 3.63 | 0.130 | 1.31 | 0.316 | K00324 | F420-non-reducing hydrogenase subunit D |
| *mttB* | 7.61 | 0.050 | 7.83 | 0.049 | 0.87 | 0.403 | K14083 | Corrinoid protein Co-methyltransferase |
| *mtmB* | 0.22 | 0.665 | 0.61 | 0.478 | 0.06 | 0.826 | K16176 |  |
| *mtbA* | 45.56 | 0.003 | 50.22 | 0.003 | 86.311 | 0.003 | K14084 |  |
| *acsA* | 25.05 | 0.013 | 30.16 | 0.042 | 48.21 | 0.040 | K00198 | Acetyl-CoA synthetase |
| *cdhA* | 8.26 | 0.045 | 1.09 | 0.355 | 0.43 | 0.545 | K00194 | CO dehydrogenase /acetyl-CoA synthase complex subunits |
| *cdhB* | 5.42 | 0.080 | 0.50 | 0.518 | 0.38 | 0.572 | K00197 |  |
| *cdhC* | 5.24 | 0.085 | 4.29 | 0.108 | 5.95 | 0.071 | K00195 |  |
| *cdhD* | 0.59 | 0.486 | 0.35 | 0.582 | 0.12 | 0.749 | K00196 |  |
| *cdhE* | 10.67 | 0.045 | 0.71 | 0.446 | 0.38 | 0.569 | K00199 |  |
| *qhqA* | 0.68 | 0.457 | 0.47 | 0.528 | 0.58 | 0.491 | K18394 | Methylamine dehydrogenase |
| *mauB* | 0.29 | 0.617 | 0.82 | 0.418 | 0.06 | 0.821 | K14082 | Methylamine dehydrogenase small subunit |
| *fmdA* | 8.70 | 0.042 | 8.83 | 0.056 | 1.52 | 0.285 | K00123 | Formate dehydrogenase subunits |
| *fdhA* | 0.65 | 0.466 | 1.06 | 0.360 | 0.24 | 0.652 | K05299 |  |
| *fdhB* | 0.95 | 0.385 | 18.42 | 0.012 | 0.58 | 0.490 | K05298 |  |
| *fdwA* | 1.16 | 0.298 | 0.68 | 0.501 | 0.13 | 0.736 | K15865 |  |
| *cooF* | 0.35 | 0.584 | 0.94 | 0.387 | 0.18 | 0.695 | K00194 | Electron transfer protein (shared with cdhA) |
| *anfG* | 12.17 | 0.025 | 0.65 | 0.466 | 0.39 | 0.565 | K04752 | Alternative nitrogenase delta chain |
| *nifH* | 78.95 | 0.001 | 1.05 | 0.363 | 0.53 | 0.509 | K02588 | Nitrogenase iron protein |
| *nifK* | 14.09 | 0.020 | 0.50 | 0.517 | 0.32 | 0.599 | K02591 | Nitrogenase molybdenum-iron protein beta chain |
| *nifD* | 10.87 | 0.030 | 0.83 | 0.415 | 0.57 | 0.494 | K02586 | Nitrogenase molybdenum-iron protein alpha chain |
| *amoA* | 9.5 | 0.044 | 15.8 | 0.024 | 10.5 | 0.039 | K10944 | Ammonia monooxygenase |
| *amoB* | 0.43 | 0.550 | 21.1 | 0.009 | 0.06 | 0.818 | K10945 |  |
| *amoC* | 8.64 | 0.042 | 10.45 | 0.032 | 2.27 | 0.206 | K10946 |  |
| *nrfA* | 15.71 | 0.017 | 0.86 | 0.407 | 0.52 | 0.510 | K03385 | Cytochrome c nitrite reductase |
| *nirB* | 13.18 | 0.022 | 0.95 | 0.384 | 0.27 | 0.628 | K00362 | Nitrite reductase (NAD(P)H) large subunit |
| *narG* | 15.22 | 0.018 | 0.43 | 0.543 | 0.61 | 0.479 | K00370 | Nitrate reductase alpha subunit |
| *nxrA* | 13.20 | 0.035 | 15.22 | 0.026 | 0.45 | 0.538 |  |  |
| *norB* | 0.63 | 0.471 | 20.53 | 0.010 | 0.42 | 0.552 | K04561 | nitric oxide reductase subunit B |
| *nosZ* | 0.50 | 0.518 | 23.33 | 0.008 | 0.72 | 0.443 | K00376 | Nitrous-oxide reductase |

**Supplementary table 3. Chemical characteristics of soil of experimental soil.**

| Year  (Miryang) | Fertilization  Level (%) | pH  (1:5) | T-N  (mg/kg) | EC  (ds/m) | Organic matters  (%) | P_2_O_5_  (mg/kg) | K  (cmolc/kg) | Ca  (cmolc/kg) | Mg  (cmolc/kg) | Na  (cmolc/kg) |
| --- | --- | --- | --- | --- | --- | --- | --- | --- | --- | --- |
| 2022 | LN (50) | 5.76 | 0.22 | 0.71 | 2.90 | 248.65 | 1.27 | 6.25 | 1.24 | 0.22 |
|  | NN (100) | 5.61 | 0.40 | 1.33 | 2.70 | 240.51 | 1.30 | 6.89 | 1.28 | 0.22 |
| 2023 | LN (50) | 5.70 | 0.21 | 0.68 | 2.82 | 258.20 | 1.22 | 6.35 | 1.21 | 0.21 |
|  | NN (100) | 5.63 | 0.42 | 1.21 | 2.73 | 242.48 | 1.29 | 6.58 | 1.25 | 0.21 |

| Year  (Jinju) | Fertilization  Level (%) | pH  (1:5) | T-N  (mg/kg) | EC  (ds/m) | Organic matters  (%) | P_2_O_5_  (mg/kg) | K  (cmolc/kg) | Ca  (cmolc/kg) | Mg  (cmolc/kg) | Na  (cmolc/kg) |
| --- | --- | --- | --- | --- | --- | --- | --- | --- | --- | --- |
| 2022 | LN (50) | 5.82 | 0.39 | 0.85 | 4.15 | 310.4 | 1.59 | 7.53 | 1.52 | 0.28 |
|  | NN (100) | 5.79 | 0.57 | 1.62 | 4.05 | 302.5 | 1.45 | 7.82 | 1.55 | 0.28 |
| 2023 | LN (50) | 5.89 | 0.32 | 0.88 | 4.20 | 315.2 | 1.62 | 6.92 | 1.50 | 0.32 |
|  | NN (100) | 5.78 | 0.60 | 1.55 | 4.10 | 305.8 | 1.58 | 7.23 | 1.58 | 0.31 |

**Supplementary table 4. Details of qRT-PCR primers used in this study**

| Primer name | Target gene | Primer sequence (5'→3') | Reference |
| --- | --- | --- | --- |
| OsINV4-F | *OsINV4* | GATACCGAGGAAGCTCTGGC | This study |
| OsINV4-R |  | GACATTGACGTGCTTGGCTC |  |
| OsHXK6-F | *OsHXK6* | CACAGCGGCGGCCGACACCACCACCGCCAC |  |
| OsHXK6-R |  | GTGGTGGTGTCGGCCGCCGCTGTGATCGAG |  |
| OsAGPL2-F | *OsAGPL2* | GGTCTGTGCAGGTATTGGCT |  |
| OsAGPL2-R |  | GCTGTACCCTGGAACCATCC |  |
| OsAPX1-F | *OsAPX1* | CATCTCCTACGCCGATTTCTAC |  |
| OsAPX1-R |  | CCTTGGTAGCATCAGGAAGAC |  |
| OsPYL1-F | *OsPYL1* | ACTGCCCCCAGGTGTACAAG |  |
| OsPYL1-R |  | ATGGTGAAGCCGAAGACGC |  |
| OsPP2C-F | *OsPP2C* | GCCGCAGCTCCGACA A |  |
| OsPP2C-R |  | CTACAGCATCAGCTG GGTGACA |  |
| OsAmy1A-F | *OsAmy1A* | GATACGACGTCGAACACCTC |  |
| OsAmy1A-R |  | CGGATCGGATACAGCTCGTTG |  |
| OsAmy3D-F | *OsAmy1D* | GATTGGGACACGGTATGACG |  |
| OsAmy3D-R |  | CTGCAGGAACTCTGAGACCG |  |
| OsIAA1-F | *OsIAA1* | GCGCTGGTGAAGGTGAGCAT |  |
| OsIAA1-R |  | ACGTACTCCAGGTCATCTCT |  |
| OsARF1-F | *OsARF1* | AATGGAAGTTGGAGATGATCCTT |  |
| OsARF1-R |  | AAGACAGTAACTTCGGGTGAGG |  |
| OsSUT1-F | *OsSUT1* | CCAAAGGGAACTGATCCTCA |  |
| OsSUT1-R |  | AAGAAACGCAAAGAGGACGA |  |
| OsMST1-F | *OsMST1* | TGACGTTCTCGGTGGTCATC |  |
| OsMST1-R |  | TAGACGCAGTACTCGTTCCC |  |
| OsSWEET11-F | *OsSWEET11* | TGGTTCTGCTACGGCCTCTT |  |
| OsSWEET11-R |  | GGTACCAGAAGTAGAGCCCCATCT |  |
| OsSUT5-F | *OsSUT5* | CGTCGCTTCTGAGGTTACTGCT |  |
| OsSUT5-R |  | CGGGTGGTGCCTTTGATG |  |
| OsGBSSI-F | *OsGBSSI* | TGGCATCCTTCGCACAAA |  |
| OsGBSSI-R |  | CCACAGGCAACCCAACTTCA |  |
| OsSSI-F | *OsSSI* | CTGATACCACGCAAGCAACC |  |
| OsSSI-R |  | AGAACAGCACAGGCGACAAA |  |
| OsMFS1-F | *OsMFS1* | CCAGCAGCACTAGAGGCAAT |  |
| OsMFS1-R |  | GTCGAACACCACTGTTGCAG |  |
| OsALMT1-F | *OsALMT1* | AGTACGAACAGCATGCAGTGAGAT |  |
| OsALMT1-R |  | TGAGTGTTTTGGCAGCTTTGATGG |  |
| OsTPS1-F | *OsTPS1* | TCAACATGAAGGTCGGGTGG |  |
| OsTPS1-R |  | AAATCAGCACAGAGCACGGA |  |
| OsPIP1-F | *OsPIP1* | AGGACCATGCCTGGAATGAC |  |
| OsPIP1-R |  | GATAGAGGGAATCGACAGATG |  |
| OsPIP2-F | *OsPIP2* | CAACGAGAAGGCGTGGCACA |  |
| OsPIP2-R |  | TTGCTTCATCAACGCCTTCCAG |  |
| OsABCG1-F | *OsABCG1* | GCAGAAGATGGACACACGGT |  |
| OsABCG1-R |  | GCAGGGTTCACATGATCTGGAC |  |
| OsHKT1-F | *OsHKT1* | GTCGAAGTTGTCAGTGCATATGG |  |
| OsHKT1-R |  | TGAGCCTCCCAAAGAACATCAC |  |
| OsAKT1-F | *OsAKT1* | GCTTCCAAAGGAAACGAGCAA |  |
| OsAKT1-R |  | GCAAGCGTATAAGCCCGTGTC |  |
| OsNRT1.1b-F | *OsNRT1.1b* | GTTTCATCATATTGGCTACC |  |
| OsNRT1.1b-R |  | ATTCTGAGACGAAGGAAGT |  |
| OsNRT1.1a-F | *OsNRT1.1a* | CAATTGGACCTATTTCGTAGCC |  |
| OsNRT1.1a-R |  | GCAGAAATGGTAAAACCCC |  |
| OsAUX1-F | *OsAUX1* | GCCCACATCCTCACCTACC |  |
| OsAUX1-R |  | GATGAACATGTTGAGCACGAA |  |
| OsEXPA17-F | *OsEXPA17* | CGCTTTGTAGATGCTGTCAATC |  |
| OsEXPA17-R |  | AGACCCTCATCACATCCTCATTATC |  |
| OsLsi1-F | *OsLsi1* | TCGCCGACTTCTTCCCTC |  |
| OsLsi1-R |  | ATCGCTCCGGTGAACTGC |  |
| OsCCaMK-F | *OsCCaMK* | AACTCACGAGGCGATGATGCTCTT |  |
| OsCCaMK-R |  | GCCTTGAACTCGTCGAAGGTGA |  |
| OsCYCLOPS-F | *OsCYCLOPS* | TGTCTTGGAGGAGCTATTTGCGGA |  |
| OsCYCLOPS-R |  | TCCGCCTTGAAACTTTGTCTGGTG |  |
| Actin11-F | *Actin1* | GCATCTCTCAGCACATTCCA |  |
| Actin11-R |  | GCGATAACAGCTCCTCTTGG |  |
| Primers used to quantify methanogenic and methanotrophic groups | | | |
| MET630F | *mcrA* | GGATTAGATACCCSGGTAGT | [9] |
| MET803R |  | GTTGARTCCAATTAAACCG |  |
| A189F | *pmoA* | GGNGACTGGGACTTCTGG |  |
| mb661R |  | CCGGMGCAACGTCYTTACC |  |
| KASP marker | | | |
| *gs3* | | Allele X : ACGCTGCCTCCAGATGCTGC | [9] |
|  |  | Allele Y: ACGCTGCCTCCAGATGCTGA |  |
|  |  | Common : AAACAGCAGGCTGGCTTACTCTC |  |

**Supplementary table 5. Details of qPCR primers used in this study**

| **Primer name** | **Gene name** | **Primer sequence (5' → 3')** | **Reference** |
| --- | --- | --- | --- |
| Mst702-F | Methanosaetaceae (Mst) | TAATCCTYGARGGACCACCA | [30] |
| Mst862-R | 16s RNA | CCTACGGCACCRACMAC |  |
| MET630-F | Methanogen (MET) | GGATTAGATACCCSGGTAGT |  |
| MET803-R | 16s RNA | GTTGARTCCAATTAAACCG |  |
| Msc380-F | Methanosarcinaceae | GAAACCGYGATAAGGGGA |  |
| Msc828-R | (Msc) 16s RNA | TAGCGARCATCGTTTACG |  |
| MBT857-F | Methanobacteriales | CGWAGGGAAGCTGTTAAGT |  |
| MBT1196-R | (MBT) 16s RNA | TACCGTCGTCCACTCCTT |  |
| MMB282-F | Methanomicrobiales | ATCGRTACGGGTTGTGGG |  |
| MMB832-R | (MMB) 16s RNA | CACCTAACGCRCATHGTTTAC |  |
| ARC787-F | Archaea (ARC) 16s | ATTAGATACCCSBGTAGTCC |  |
| ARC1059-R | RNA | GCCATGCACCWCCTCT |  |
| MCL282-F | Methanocella-specific | ATCMGTACGGGTTGTGGG |  |
| MCL832-R | (Met) 16s RNA | CACCTAGCGRGCATCGTTTAC |  |

**Supplementary Table 6. List of key differentially expressed genes (DEGs) in rice panicle and root tissues**

List of key genes showing differential expression in the panicle and root tissues of LN-Milyang360 compared to NN-Saeilmi. The Log₂ fold change and adjusted p-values were determined from the RNA-seq data analysis using DESeq2.

| Tissue | Functional Category | Gene | Gene Description | Log2 Fold Change | Adjusted *P* -value |
| --- | --- | --- | --- | --- | --- |
| Panicle | Gluconeogenesis | *OsINV4* | Invertase 4 | -2.5 | 6.E-11 |
|  |  | *OsHXK6* | Hexokinase 6 | -2.1 | 9.E-06 |
|  |  | *OsPEPCK1* | Phosphoenolpyruvate carboxykinase 1 | -0.9 | 3.E-07 |
|  | Carbon reservoir activity | *OsTPS1* | Trehalose-6-phosphate synthase 1 | -2.5 | 4.E-05 |
|  |  | *OsAGPL2* | ADP-glucose pyrophosphorylase large subunit 2 | -1.8 | 5.E-04 |
|  | Oxidoreductase | *OsAPX1* | Ascorbate peroxidase 1 | 2.3 | 2.E-03 |
|  |  | *OsPOD1* | Peroxidase 1 | 1.9 | 1.E-03 |
|  |  | *OsCATA* | Catalase A | 1.2 | 3.E-02 |
|  |  | *OsCSD1* | Cytosolic Copper/Zinc Superoxide Dismutase 1 | 1.6 | 4.E-02 |
|  | ABA signaling | *OsPYL1* | Abscisic acid receptor PYL1 | -1.5 | 2.E-03 |
|  |  | *OsPP2C* | Protein phosphatase 2C | -1.3 | 8.E-03 |
|  | Starch degrading activity | *OsAmy1A* | Alpha-amylase 1A | -2.7 | 6.E-08 |
|  |  | *OsBAM2* | Beta-amylase 2 | -2.3 | 6.E-04 |
|  | Auxin signaling | *OsIAA1* | Auxin-responsive protein IAA1 | 1.5 | 2.E-03 |
|  |  | *OsARF1* | Auxin response factor 1 | 1.5 | 2.E-03 |
|  |  | *OsSnRK2.6* | Stress-Activated Protein Kinase 2 | 1.3 | 3.E-03 |
|  |  | *OsbZIP23* | bZIP Transcription Factor | 1.2 | 4.E-02 |
|  |  | *OsTRAB1* | Transcription Factor 1 | 1.2 | 5.E-02 |
|  | Carbohydrate transporter | *OsMST1* | Monosaccharide transporter 1 | 1.7 | 3.E-03 |
|  |  | *OsMST6* | Monosaccharide Transporter 6 | 1.5 | 5.E-03 |
|  | Sugar transporter | *OsSUT5* | Sucrose transporter 5 | 3.5 | 7.E-09 |
|  |  | *OsSWEET11* | Sugar transporter SWEET11 | 2.6 | 2.E-03 |
|  |  | *OsSUT1* | Sucrose transporter 1 | 2.5 | 7.E-06 |
|  |  | *OsSWEET14* | Sugar transporter SWEET14 | 1.8 | 7.E-05 |
|  |  | *OsSWEET15* | Sugar transporter SWEET15 | 1.5 | 3.E-04 |
|  |  | *OsSUT2* | Sucrose Transporter 2 | 1.0 | 3.E-02 |
|  | Starch biosynthesis | *OsSSI* | Starch synthase I | 1.9 | 8.E-04 |
|  |  | *OsGBSS1* | Granule-bound starch synthase I | 1.7 | 1.E-05 |
|  |  | *OsISA1* | Isoamylase 1 | 1.5 | 3.E-03 |
|  |  | *OsBEIIb* | Starch Branching Enzyme Iib | 1.5 | 5.E-03 |
|  |  | *OsSSIIa* | Starch Synthase Iia | 1.2 | 1.E-02 |
| Root | Carbon transporter | *OsSUT1* | Sucrose transporter 1 | -5.3 | 3.E-04 |
|  |  | *OsMST8* | Monosaccharide transporter 8 | -5.1 | 8.E-04 |
|  |  | *OsSWEET11* | Sugar transporter SWEET11 | -3.5 | 3.E-03 |
|  |  | *OsSWEET4* | Sugar transporter SWEET4 | -2.0 | 9.E-03 |
|  |  | *OsSUT4* | Sucrose Transporter 4 | -1.8 | 1.E-02 |
|  |  | *OsMST3* | Monosaccharide Transporter 3 | -1.5 | 1.E-02 |
|  | Organic acid transporter | *OsALMT1* | Aluminum-activated Malate Transporter 1 | -5.1 | 3.E-03 |
|  |  | *OsMFS* | MFS transporter | -4.0 | 3.E-03 |
|  |  | *OsALMT4* | Aluminum-activated Malate Transporter 4 | -2.5 | 9.E-03 |
|  |  | *OsFRDL4* | Ferric Reductase Defective Like 4 | -1.9 | 3.E-02 |
|  | Alcohol transporter | *OsABCG1* | ABC transporter G family member 1 | -3.6 | 3.E-03 |
|  |  | *OsPIP2* | Aquaporin PIP2 | -3.5 | 1.E-03 |
|  |  | *OsTIP1.1* | Tonoplast Intrinsic Protein 1.1 | -2.0 | 4.E-03 |
|  |  | *OsNIP3.1* | Nodulin-26 like Intrinsic Protein 3.1 | -1.5 | 2.E-02 |
|  | Nitrate transporter | *OsNRT1.1b* | Nitrate transporter 1.1b | 7.5 | 3.E-04 |
|  |  | *OsNRT2.1a* | Nitrate transporter2.1a | 3.1 | 4.E-06 |
|  |  | *OsNRT2.2* | Nitrate Transporter 2.2 | 2.0 | 6.E-03 |
|  |  | *OsNAR2.1* | Nitrate Assimilation-Related 2.1 | 1.9 | 5.E-04 |
|  |  | *OsNPF2.4* | NRT1/PTR FAMILY 2.4 | 1.5 | 6.E-03 |
|  | Ammomium transporter | *OsAMT1.1* | Ammonium Transporter 1.1 | 9.0 | 7.E-06 |
|  |  | *OsAMT2.1* | Ammonium Transporter 2.1 | 1.5 | 6.E-03 |
|  |  | *OsAMT1.3* | Ammonium Transporter 1.3 | 1.5 | 3.E-03 |
|  |  | *OsAMT3.2* | Ammonium Transporter 3.2 | 1.3 | 6.E-03 |
|  | Root hair elongation | *OsEXPA17* | Expansin-A17 | 4.9 | 2.E-04 |
|  |  | *OsAUX1* | Auxin influx carrier 1 | 4.0 | 4.E-03 |
|  |  | *OsEXPA30* | Expansin-A30 | 3.0 | 5.E-03 |
|  |  | *OsRSL4* | ROOT HAIR DEFECTIVE SIX-LIKE 4 | 1.5 | 4.E-02 |
|  |  | *OsRBOHC* | Respiratory Burst Oxidase Homolog C | 1.3 | 4.E-02 |
|  |  | *OsRHL1* | ROOT HAIRLESS-LIKE 1 | 1.2 | 3.E-02 |
|  | Apoplast pathway | *OsPIP1* | Aquaporin PIP1 | 1.9 | 3.E-02 |
|  |  | *OsLsi1* | Silicon influx transporter 1 | 2.5 | 3.E-02 |
|  |  | *OsLsi2* | Silicon influx transporter 2 | 1.3 | 5.E-02 |
|  | Symbiosis pathway | *OsCYCLOPS* | CYCLOPS protein | 5.8 | 2.E-05 |
|  |  | *OsCCaMK* | Calcium/calmodulin-dependent kinase | 5.0 | 3.E-04 |
|  |  | *OsCERK1* | Chitin Elicitor Receptor Kinase 1 | 1.1 | 1.E-02 |
